# A Study of Zero Anaphora Resolution in Chinese Discourse: From the Perspective of Psycholinguistics

**DOI:** 10.3389/fpsyg.2021.663168

**Published:** 2021-10-28

**Authors:** Ning Yang, Jingyu Zhang, Lijun Ma, Zhi Lu

**Affiliations:** ^1^School of Education, South China Normal University, Guangzhou, China; ^2^School of Interpreting and Translation Studies, Guangdong University of Foreign Studies, Guangzhou, China; ^3^School of Public Health and Management, Guangzhou University of Chinese Medicine, Guangzhou, China; ^4^Center for Translation Studies, Guangdong University of Foreign Studies, Guangzhou, China

**Keywords:** zero anaphora resolution, psychological reality, time course, topic continuity, situation consistency

## Abstract

Anaphora is the main linguistic means to establish discourse coherence, and anaphora resolution is the psychological process to maintain this coherence. Chinese discourse is characterized with providing multiplicity of linguistic clues to readers by employing various referential apparatuses such as pronoun anaphora, zero anaphora, and so on. As a way of avoiding repeated reference to an object that is mentioned beforehand, zero anaphora is frequently employed in discourse. The production and resolution of zero anaphora largely concerns some constraints underlying psychological mechanisms. We particularly focus on zero anaphora resolution in the present study to try to discover some specific aspects of the underlying mechanism, hoping to find out some factors unique to the resolution process. We designed the first two experiments to probe into the psychological reality when participants were presented with sentences containing either pronoun anaphora or zero anaphora or both under discourse condition with topic continuity in Experiment 1a and topic discontinuity in Experiment 1b. We did not find any significant difference in the reaction time between zero anaphora resolution and pronoun anaphora resolution, indicating that zero anaphora possibly works within the processing mechanism on which pronoun anaphora resolution depends. However, we found significantly longer time in reading the first sentence in any of the discourse, showing that the first-mention effect exists in anaphora resolution. We further explored the time course of zero anaphora resolution by measuring the reaction time during the period when participants read sentences that varied according to the location where zero anaphora occurred under two conditions: topic continuity (Experiment 2a) vs. topic discontinuity (Experiment 2b). The strategies of searching for the referential information were found divergent: the exhaustive searching strategy was adopted when the topics within a discourse were kept continuous and the heuristic searching strategy was employed when the topics were discontinuous. The design of Experiment 5 took the factor of voice type and situation consistency into consideration, investigating in what way do those factors influence the resolution of zero anaphora. The voice type, according to the results, plays a significant role for its exclusively close relationship with the first-mention effect.

## Introduction

Anaphora is interpreted divergently. From the perspective of traditional grammar, it is described as a grammatical relationship among linguistic elements, for example, Crystal (2008) thinks that anaphora is used to “refer to some other sentence constituent.” Anaphora is also considered as a grammatical device created for the purpose of avoiding the repetition of a prior mentioned information. Scholars of pragmatics (such as Levinson), however, hold the opinion that anaphora is the identity of entities (e.g., Hirst, 1981). For any entity that is designated as the “correct” one, there is a choice from a set of possible anaphoric expressions during the dynamic course of discourse production. This brings the anaphora into the scope of keeping the topic continuous during discourse comprehension and establishes the theoretical basis on which the anaphora is analyzed from the perspective of pragmatics. Huang (2000) further develops the pragmatic model, known as the neo-Gricean pragmatic approach, by employing some basic pragmatic strategies such as Levinson's Q-, I-, and M-principles, and proposes that those pragmatic principles account for the anaphora referent. Despite some minor differences, the pragmatic models believe that the anaphora plays a role as a substitution of the structural constituent and regard the reference and its referent as co-reference. The key fact is that how the anaphoric or co-referential relationship is established in mind and in what way do people to resolve the anaphora. Indeed, the anaphora resolution is “the process by which an antecedent is assigned to an anaphora” (McDonald and MacWhinney, 1995). On the one hand, from the perspective of anaphoric production, people are instinctive to avoid information redundancy, so they prefer to use reference to achieve this goal. On the other hand, from the vantage point of anaphoric resolution, listeners or readers have to develop the ability of identifying the possible referent and resolve it in order to understand the information. The two perspectives boil down to this: the construction and resolution of anaphora largely depends on anaphora distribution, which is a complex phenomenon involving structural, cognitive, and pragmatic factors that interact with each other (Huang, 2000). The question arises accordingly that how to examine the factors that take effect in exploring anaphora resolution. With the increasing number of the empirical studies, a lot of approaches have been proposed to try to answer the above question, such as the topic continuity or distance-interference model (Givón, 1983), the hierarchy model (Hinds, 1978, 1979; Tai, 1978; Longacre, 1979), and the cognitive model (Gundel et al., 1993; Lambrecht, 1994; Kibrik, 1996). The views are unsystematic and inconsistent because they mainly concern discourse processing. Anaphora is mentioned very briefly and discussed in a rather general way.

In fact, anaphora is essentially a way of reference to entities mentioned earlier in discourse. The discourse comprehension largely relies on resolving references used within the text with the same sense (i.e., referring back to the same entity) (Sukthanker et al., 2020). Anaphora resolution is thought complicated particularly when it appears in a discourse rather than in a sole sentence either in terms of the linear distance between the two mentions of referent or in terms of the number of interfering referents. Additionally, the different anaphoric forms that “reference” is resembled as also differentiate the difficulty of resolution: compared with zero anaphora resolution, pronoun anaphora resolution is supposed much easier since there is a tangible form of pronoun occurring in the place where anaphora is necessary. For example, in the sentence “*Alex (1) ran into her (2) room*,” (2) is the pronominal referent of (1). The referent in this sentence is presented with the antecedent “Alex” with the pronominal form “her,” leading the reader to a correct identification of the relationship between the anaphora and its referent. Ariel (1990) believes that different forms of reference in discourse is to refer to different accessibility of referential entities, thus, he divides the degree of accessibility into three categories and points out, for example, in deictics, the distal (such as “that”) are less accessible than the proximal (such as “this”).

The study of anaphora not only involves solving problems of the anaphoric phenomenon, but also relates to establishing, retaining, and shifting of topic continuity, which, in turn, matters the discourse coherence. When we examine the example further, we find that the topic continuity also contributes to the fast understanding of the sentence by people, because the pronoun “her” serves as a clue for the reader to connect one topic “Alex” with another topic “room” together to keep the topic continuous. Contrary to the tangible form of pronoun anaphora, zero anaphora does not have a visible lexical and phonetic form. For example, in the sentence “*You have two choices (1): to stand still (2) vs. to move forward (3)*,” phrases (2) and (3) refer back (are anaphoric) to the same phrase (1). The difficulty of resolving zero anaphora lies on the fact that there is no interfering referent to clearly indicate the anaphora relationship. This arises an important question “how to avoid the arbitrariness in the confirmation of anaphora when it does not appear physically in the discourse.” One of the most involved type of encoding a referent is to evaluate with a short distance to its previous mention.

Zero anaphora is rarely seen in English, but frequently adopted as a way to refer back to previously named entities in Chinese. The reasons might be as follows: at first, compared with English, the sentence-oriented and subject-prominent language Chinese is more topic-prominent and discourse-oriented, requiring attention to attaining the topic within a macro discourse level rather than a sentence level. In another word, Chinese is what Song (2003) called the parataxis language, which is characterized with constructing the discourse coherence with analyzing the scattered grammatical components followed by discovering the semantic and internal logic among them. The evidence from Chinese narrative discourse shows that 91.3% of the Chinese zero anaphora have distributed within the same sentence and 93.4% of them are manifested as subjects of the sentence (Hou and Sun, 2005). Li and Thompson (1979) also prove that zero anaphora in Chinese is far more extensively used than that in English. Next, in many cases, there is typically one referent that is established by a given topic but shared by other unrealized topics among the main clause and its subordinates. This type of zero anaphora is called “topic chain,” which is frequently occurring in Chinese discourse in order to keep topics continuous. The topic referent is distinguished from the non-topic referent according to whether the anaphoric form is a reduced anaphoric form or a non or less-reduced anaphoric form. Meanwhile, a short referential distance and no interfering referent also affect the choice of topic referent, particularly in some East Asian languages. Thinking deeply about the distance-interference effect, the process of highlighting topic referent and inhibiting non or less-topic referent is rooted from the cognition of people (e.g., Tomlin and Pu, 1991) or pragmatic principles (e.g., Huang, 1989, 1994). Chinese, a thematic-prominent language, is particularly governed by the principles. On the one hand, the use of zero anaphora largely relies on pragmatic and textual knowledge (Zhang and Cui, 2001; Li, 2004). On the other hand, the repetition of verbs, the gradation of clauses, and the use of other types of anaphora probably cause topic discontinuity. Moreover, the introduction of more than one topic in discourse causes mental competition when people identify the topic referent by ruling out the possibility of the non or less-topic referent.

In Chinese, the referential relationship is not exclusively recognized by zero expression, but by pronouns (like *him*), full-noun phrases (like *Alex* or *the boy*), and reflexives (like *ziji*). Pronoun anaphora and noun anaphora are most commonly discussed in literatures, the notorious reflexive *ziji* (or “self”), however, attracts more and more attention. According to the theoretical linguistic literature, the use of reflexive illustrates the binding relationship (e.g., Huang, 1982; Mohanan, 1982; Wang and Stillings, 1984) and the discourse co-reference with its antecedents (e.g., Yu, 1991; Huang, 1994; Huang and Liu, 2001). The psycholinguistic researchers (e.g., Carroll, 1999; Gao et al., 2005; Liu, 2009) examined the cognitive process of identifying the referent of the Chinese reflexive *ziji* and multiple occurrences of the Chinese reflexive *zijis*. The binding effect (e.g., Carroll, 1999; Liu, 2009), the local-binding effect (e.g., Gao et al., 2005), and the long-distance binding effect (e.g., Shuai et al., 2013) are found. The ERP (Event-Related Potential) is an electrical brain response, recorded via electroencephalography, time-locked to the onset of an event such as a sensory stimulus or a motor act experiment, conducted by Li and Zhou (2010), confirms that the selection of a matrix subject as the long-distance antecedent of *ziji* violates the Principle A during the comprehension of sentences and requires more mental resources. The findings have revealed that some aspects concerning the mechanism above which Chinese reflexive is processed within a range of syntactic distance, the mental resources involvement and the time course. The use of reflexive relates to the understanding of the entity referents by people. Even though it shares similarities with zero anaphora, i.e., forming not only a referential relationship but also a distribution relationship, reflexive anaphora is quite different from zero anaphora because reflexive anaphora, in one way or another, mainly plays a role of intensifying the antecedent (like *Wo ziji*), functioning more as an adverb in grammar rather than the reference of the antecedent. *Z*ero anaphora, however, does not have such a portrait.

Chinese scholars have done extensive studies on zero anaphora either from the perspective of textual analysis (e.g., Lv, 1984; Xu, 1992; Tao and Healy, 2005) or from the pragmatic perspective (e.g., Xiong, 1999). Most of them, if not all, have reached the following consensus: (1) zero anaphora is more frequently used both in written and oral Chinese than it is used in English; (2) due to less strict grammatical rules, the location where zero anaphora occurs is flexible; (3) in some cases (e.g., job interview), zero anaphora is realized without the employment of antecedents; (4) in most cases, the use of zero anaphora is restricted by pragmatic and psychological factors; and (5) certain pronouns and conjunctions supply premise for the use of zero anaphora. These claims are mainly rooted from analyzing the linguistic features that Chinese zero anaphora possesses when it occurs under a particular circumstance on the basis of comparing it with other types of anaphora. However, there are some very important issues the claims do not explain well. For example, in such a case in which anaphors appear within the same clause as their antecedent, there seem to be strong syntactic constraints on the range of possible anaphoric forms. Comparing the two sentences “我喝了 柠檬水,Φ很舒服 ” and “我喝了 柠檬水,Φ很酸, ” we usually refer back to the antecedent of “我 ” (the subject of the first clause) in the first sentence, but of “柠檬水” (the object of the first clause) in the second sentence. The difference of the accessibility of more than one antecedent in the same discourse indicates that zero anaphora is discrete in space which is not merely governed by syntactic rules. It is assumed that there might be an underlying mechanism beyond the rules that are responsible for the resolution of zero anaphora.

To our knowledge, the majority of studies on Chinese anaphora resolution examined the relationship between pronoun anaphora and discourse comprehension, that is, in what way and in which aspects pronoun anaphora is influenced during the reading comprehension (e.g., Miao, 1994, 1996a,b; Miao and Song, 1995; Wang and Li, 1999; Sun et al., 2001; Zhou et al., 2001; Bai et al., 2005; Jiao and Zhang, 2005; Shen and Yang, 2006; Wang et al., 2006). Instead of paying close attention to anaphora itself, the studies focused on comparing reading strategies and comprehending abilities of processing materials provided in Chinese and a second language. Only a small number of studies painted a mixed picture of Chinese anaphora resolution in discourse processing (e.g., Wang and Mo, 2001; Zhao and Liu, 2006; Zhao and Mo, 2007). In spite of the rich achievements, most of the studies are restricted within the area of Chinese pronoun anaphora and its resolution. Research of zero anaphora was sporadic and unsystematic with little consideration from the psycholinguistics perspective. That is what we mainly concern in the present study: the psychological mechanism with which people can access the antecedent successfully without the indication of obvious referent. Additionally, since Givón (1979, 1995) repeatedly mentioned that topic continuity is the most important textual condition in affecting the use of zero anaphora, we took it into consideration when designing our experiments by addressing the existence and absence of topic continuity in constructing experimental materials.

## The Present Research

### Research Questions

The convergent idea is that, compared with pronoun anaphora and noun anaphora, the referent of zero anaphora is more difficult to identify. For example, when we encounter pronouns or repeated nouns in a discourse, we often automatically start to search the antecedent by referring back to the recent NP. Conversely, we can't follow the same route if we meet a sentence where zero anaphora is contained because there is a lack of tangible form used to encode a referent to its previous mention. In other words, unlike pronoun anaphora and noun anaphora, zero anaphora fails to be supported either by language form (e.g., *it* in pronoun anaphora) or by lexical meaning (e.g., *noun* or *noun phrase* in noun anaphora), or even sometimes by its antecedents (e.g., no antecedent). Accordingly, zero anaphora requires more mental resources than the other two types of anaphora and might be resolved in a way unique to others. We are wondering what mental activities are involved if we resolve zero anaphora during reading comprehension. Previous studies have proved that when anaphora resolution starts, not only an antecedent but also several competing antecedents and concepts co-occurring with them are activated as candidates (Corbett and Chang, 1983; Dell et al., 1983; Gernsbacher et al., 1989; Greene et al., 1992). Only those entities that are first mentioned with a grammatical marker in one way or another are considered closely related to the topic and are highly activated. The activation begins 250 ms after the presentation of the anaphora ends. Dell et al. (1983) used probe word insertion technique to explore to what extent the activation is achieved in noun anaphora resolution and find that both the antecedent of noun anaphora and its co-occurring concepts are activated but only the antecedent keeps being activated as the location of the probe word changes from one place to another. We are wondering in what way the zero anaphora is resolved and when the activation of the antecedent begins during the resolution. We are also curious about the factors related to the resolution of zero anaphora on the basis of existing studies, in which factors concerning pronoun anaphora resolution are summarized into the following categories: (1) the linear distance between the pronoun and its antecedent, (2) the causation implicitly contained in verbs, and (3) the first-mention effect on the accessibility of the pronoun antecedent.

### Research Hypotheses

#### Hypothesis 1

With the same level of activation, the response time to the probe word in zero anaphora resolution is as fast as it is in pronoun anaphora resolution, no matter the topic is continuous or not. However, the reading time of sentences containing pronoun anaphora is longer than those containing zero anaphora and normal sentences for the ambiguous reference in pronoun anaphora examples.

#### Hypothesis 2

Being that there is a lack of tangible form of zero anaphora, people might use different strategies to cope with the situations in which the probe word is inserted in different positions: either right after the verb or at the end of the sentence. We expect that if the probe word is inserted after the verb, people use a heuristic strategy to search for the antecedent. If the probe word is inserted at the end of the sentence, people use an exhaustive strategy to search.

#### Hypothesis 3

The factors of situation consistency and voice type are critical to zero anaphora resolution. We expect that response time to the probe word is the fastest when the consistent situation is described in an active voice whereas the response time is the slowest when the inconsistent situation is described in a passive voice.

### Experiment 1

This experiment adopted the research method from Gernsbacher (1989) to explore the psychological reality of zero anaphora resolution by measuring response time to the probe word under the condition of topic continuity and topic discontinuity. The materials were classified into three types, each of which contained one of the anaphors: pronoun anaphora, zero anaphora, and both pronoun and zero anaphora.

#### Experiment 1a: The Psychological Reality of Zero Anaphora Resolution Under the Condition of Topic Continuity

##### Subjects

A total of 20 sophomores with normal or corrected-to-normal vision took part in this experiment. They were all Chinese native speakers.

##### Materials and Design

Fifty-four short essays were made up as the experimental materials were made up of fifty-four short essays and fell into 18 groups randomly, each of which involved three types of anaphora named as A for pronoun anaphora, B for zero anaphora, and C for both. Each essay consisted of five sentences^1^. All the essays shared the same topic as well as the same sentence structure. Type C essays were the control materials that had been evaluated with the 5-point Likert Scale by juniors majoring Chinese Literature before the experiment and those rated above 4 were selected. The examples were shown below:

A /中午李明到了学校/接着她开始上课/课后她又到同学那里串门/她坐到快天黑时/她才恋恋不舍地告别回家了/ (pronoun anaphora)
B /中午李明到了学校/接着Φ开始上课//课后Φ又到同学那里串门/Φ坐到快天黑时/Φ才恋恋不舍地告别回家了/ (zero anaphora)
C /中午李明到了学校/接着她开始上课//课后Φ又到同学那里串门/Φ坐到快天黑时/她才恋恋不舍地告别回家了/ (control material)

We adopted an offset equilibrium method, similar to the Latin square for the purpose of avoiding the familiarity effect. Specifically, we divided all participants into 6 groups, giving the first group type A material and used true antecedents as probe words (e.g., 李明). The second group was given type B material with false antecedents as probe words (e.g., 王亮) and so on. The experiment was a 3 (type of anaphora: A/B/C) × 2 (probe word: true /false antecedent) within-group design.

##### Procedure

The experiment was implemented by using E-prime software. Prior to the experiment, the signal “+” was first displayed on the screen for 600 ms, followed by a sentence presented character by character (a moving window technique) in the way in which the first character “中” of the first clause “中午李明到了学校” was presented on the screen for 300 ms and the next character followed and stayed for another 300 ms. They did not disappear until the last character “校” was given for 300 ms. After that, the next clause “接着开始上课” began to appear on the screen in the same way. When the last character of the whole sentence “了” disappeared and the screen was blank for a short period of time, and a probe word, either true to the discourse like “李明” or false like “王玲” appeared and lasted for 3 s. Participants were asked to judge as quickly as possible whether the probe word was previously presented. If participants made wrong judgments, a red sign was presented on the screen. Six hundred millisecond later, they had to answer a question to test their comprehension ability. The question lasted for 800 ms and feedback was given when the answer to the question was incorrect. All participants received a question after one trial to ensure that they had read the essay carefully. The procedure needed practicing beforehand.

##### Results

We deleted three extremes which were <1% of the total. The average reaction time has been listed in Table 1. Analysis of variance was used to reveal the interaction effect between the anaphora type and the probe word type. The difference was found insignificant with *F*_(2, 38)_ = 0.348, *p* > 0.05. The main effect of the reaction time to probe word type under three conditions was not significant [*F*_(2, 38)_ = 1.708, *p* > 0.05]. The main effect of probe word type was also not significant [*F*_(1, 38)_ = 1.7758, *p* > 0.05].

**Table 1:** Average reaction time to two types of probe words on the basis of the stimuli varied in different anaphora types under the condition of topic continuity in Experiment 1a (ms).

**Probe word type**	**Anaphora type**		
	**Pronoun anaphora**	**Zero anaphora**	**Control material**
True antecedent	886.23	873.6	790.51
False antecedent	914.63	877.63	843.28

The reading time of each clause was presented in Table 2. The ANOVA analysis (two-way repeated measures) showed that there was no interaction effect between sentence order and anaphora type [*F*_(8, 112)_ = 1.698, *p* > 0.05]. The main effect of sentence order was significant [*F*_(4, 56)_ = 78.551, *p* < 0.001], indicating that the reading time of each sentence varied in accordance with the sentence order: participants took the longest time in reading the first sentence. But the reading time was not significantly different among the rest of the sentences. We did not find the main effect of anaphora type [*F*_(2, 28)_ = 2.814, *p* > 0.05] showing that anaphora type did not produce any difference in reading time for participants.

**Table 2:** Average reading time of each clause within sentences that are varied in anaphora types under the condition of topic continuity in Experiment 1a (ms).

**Anaphora type**	**The order of clause**				
	**1st clause**	**2nd clause**	**3rd clause**	**4th clause**	**5th clause**
Type A	1,869.75	1,240.6	1,069.89	943.83	1,048.01
Type B	1,999.87	1,174.98	992.89	945.81	963.42
Type C	1,964.88	1,069.15	920.86	852.11	875.87

#### Experiment 1b: Psychological Reality of Zero Anaphora Resolution Under the Condition of Topic Discontinuity

##### Subjects

Another forty sophomores with normal or corrected-to-normal vision took part in this experiment. They were all Chinese native speakers.

##### Materials and Design

The experiment materials were composed of 12 groups of essays with each group containing two types of essays. Type A Essay contained zero anaphora only and type B contained both pronoun anaphora and zero anaphora. For each group, type B essays were compiled by matching the topic and the number of sentences with type A essay and being evaluated on the Likert Scale by the same group of students in Experiment 1a. All type B essays were rated above 4 and were used as control materials. The examples were shown below:

A /林冰正在塘边放鸭子/Φ1养的鸭子个个又肥又大/突然Φ1看见几个小孩赤身露体/Φ2一起在池塘追逐打闹/Φ1便大声喊要他们小心/ (zero anaphora)
B /林冰正在塘边放鸭子/她养的鸭子个个又肥又大/突然她看见几个小孩赤身露体/Φ2一起在池塘追逐打闹/她便大声喊要他们小心/ (control material)

We adopted the same method, an offset equilibrium method, in designing this experiment. We allocated all the subjects into four groups and each group only did the experiment under one experimental condition. For example, the first group of participants were presented with type A essay followed by “林冰” (true antecedent as the probe word) and the second group with type B essay followed by “李明” (false antecedent as the probe word). The experiment adopted 2 (type A essay/type B essay) × 2 (true antecedent/false antecedent) within group design.

##### Procedure

The experiment was implemented following the same procedure in Experiment 1a.

##### Results

We deleted five extreme data which were <5% of the total and the rest were listed in Table 3. We used a two-way repeated measures ANOVA on reaction time to examine the interaction effect of essay type and probe word type. The interaction effect was not significant [*F*_(1, 39)_ = 0.687, *p* > 0.05] nor was the main effect of probe word type [*F*_(1, 39)_ = 0.624, *p* > 0.05], indicating that the participants did not show any difference in reading the two types of essays. The insignificant main effect of probe word type [*F*_(1, 39)_ = 0.657, *p* > 0.05] revealed that regardless of the probe word type, the reaction time was not affected.

**Table 3:** Average reaction time to two types of probe words on the basis of the stimuli varied in different anaphora types under the condition of topic discontinuity in Experiment 1b (ms).

**Probe word type**	**Anaphora type**	
	**Zero anaphora**	**Control material**
True antecedent	873.6	790.51
False antecedent	877.63	843.28

The reading time of each clause was presented in Table 4. An ANOVA was performed for the design of 2 × 2 two-factor repeated measurement of the reading time in Experiment 1b (Table 4). The results showed that the interaction between sentence order and essay type was not significant with *F*_(4, 156)_ = 2.079, *p* > 0.05. The main effect of sentence order was significant [*F*_(4, 156)_ = 101.467, *p* < 0.001]. The reading time of the first sentence was comparatively longer than that of the rest sentences. The main effect of essay type was not significant [*F*_(1, 39)_ = 0.739, *p* > 0.05].

**Table 4:** Average reading time of each clause within sentences that are varied in anaphora types under the condition of topic discontinuity in Experiment 1b (ms).

**Anaphora type**	**The order of clause**				
	**1st clause**	**2nd clause**	**3rd clause**	**4th clause**	**5th clause**
Type A	3386.05	2147.4	2014.57	1585.33	1492.28
Type B	3278.47	1935.74	1887.38	1589.24	1500.11

#### Discussion of Experiment 1a and 1b

Whether the topic was continuous or not, participants showed non-significant difference in reaction time during zero anaphora resolution. This was consistent with our hypothesis. We assumed that this insignificant role topic continuity played in discourse comprehension was due to the same degree of topic availability to readers. By closely examining the two sentences “ /中午李明到了学校/……Φ才恋恋不舍地告别回家了/ (zero anaphora)” vs. “/林冰正在塘边放鸭子/……Φ1便大声喊要他们小心/ (zero anaphora),” we found that the topic of the first clause were easily referred back no matter whether they were the true antecedent or not. Even though the topic was not kept continuous, participants would also identify the antecedent to the first topic they encountered. The low demand of topic availability did not produce any significant differences in the reaction time. Wang et al. (2001) also proved that the anaphora could be resolved even when the antecedent was not correct to the anaphora. However, the anaphora type also did not exert any significant difference on reading time. We contributed the reason to the existence of more than one pronoun that misled people and caused ambiguity in resolving anaphora, so that the repetition of the same pronoun interrupted the smooth reading and forced participants to read back to catch up with the meaning and refer back to determine what the pronoun stood for. It is worth mentioning that probe words both in zero anaphora resolution and in pronoun anaphora resolution were activated at the same level, which was reflected in the insignificant reaction time in our experiment. In fact, according to Greene et al. (1992), the resolution of pronoun anaphora in discourse reading occurs automatically without being realized by people. Combined with our research results, we could imply that resolving zero anaphora might share the same underlying mechanism with resolving pronoun anaphora. However, it was contradictory to what Huang, 2001 pointed out: pronoun anaphora contains semantic meaning that is more transparent than zero anaphora does. In other words, zero anaphora was more difficult to resolve than pronoun anaphora.

In fact, Chinese language learners from foreign countries showed their avoidance of using zero anaphora (Xu and Xiao, 2008; Zhou, 2011). In contrast, Chinese native speakers preferred to utilize zero anaphora to indicate the referential relationship, as much as 36% of the total anaphora application (Kim, 2000). The difference might be caused by the strategy that was transferred from resolving pronoun anaphora to resolving zero anaphora within one language or among languages (Tao and Healy, 2005). But our results spoke against the theory proposed by Kintsch et al. (1999), who claimed that the thesis (argument overlap) is a key factor in maintaining coherence where there is the anaphora. The realization of argument overlap relied on the occurrence of noun phrase rendering, which was absent in zero anaphora. At this point, reading time should be increased, but we did not observe it no matter if the anaphora was pronominal or zero.

What we observed was that in spite of the different essay types, it took the participants significantly longer time in reading the first sentence than reading other sentences in both experiments. In fact, the first sentence played a role in building a foundation for comprehending the rest part and applying clues for combing the description of previous sentences and subsequent sentences. It required a lot of cognitive effort. “These initial segments help them establish the basis for the mental representation of larger units, such as sentences, paragraphs, and storylines” (Gernsbacher and Foertsch, 1999). As a result, more time was consumed. Other studies, such as Anderson (1983, 1985), and Anderson and Sanford (1983), also proved that the reading time of the first sentence was systematically and significantly longer than that of the other sentences.

We expected the increased reading time at those places where the topic of the discourse had been transferred, but we failed. The reason might be due to the materials, which were made up through inserting a sentence intentionally to stop the topic for a while instead of changing the topic into a new one. For example, 王亦东推了自行车进了门, Φ瞧见李贵在刷油漆,他的老伴儿陪在一旁给打扇子, and Φ真是从心里羡慕 (c.f. Liu, 2001). Chen (1987) believed that the semantic structure of the sentence has two characteristics: on one hand, the parenthetical sentence is subordinate to either the sentence where the antecedent occurs or that where the anaphora occurs. In our example, “李贵在刷油漆,他的老伴儿陪在一旁给打扇子” was the object clause of “瞧见,” which was lower in the level than the main sentence where the antecedent and the anaphoric object occurred. On the other hand, the grammatical structure of the inserted sentence sometimes produced ambiguity in meaning, so it was important to avoid being complicated and redundant in using zero anaphora. As it was indicated in another example, “张宁匆匆忙忙赶到火车站, Φ1庆幸总算赶上火车了, Φ1把车票递给正在检票的列车员, Φ2说票是假的, and Φ1一时觉得有点不知所措,” the meaning where “Φ 2” located was ambiguous because either “列车员” or “张宁” grammatically fit into the sentence. Thus, zero anaphora here was not acceptable unless we changed the sentence into “……列车员说票是假的, 张宁一时觉得有点不知所措.”

### Experiment 2

Previous studies have shown that the resolution of pronoun anaphora is heuristic, showing that the activation of searching for the antecedent begins as soon as the pronoun occurs. However, the resolution of zero anaphora does not possibly fit into the situation. Instead of being introduced by pronouns, there is a lack of the anaphora in zero anaphora, which increases the difficulty of referring back to the antecedent, particularly when the candidate of the antecedent is more than one. Examining the following example “小吴不但接受了李明的帮助, 而且Φ立即表示愿意改正错误(zero anaphora),” we infer that if people begin to search for the antecedent immediately after the verb “表示” shows, they probably resolve the anaphora in a heuristic way. Otherwise, they do in an exhaustive way if the search begins after the last character (e.g., “错误”) is given. Consequently, the probe word after the verb “表示” should receive the highest level of activation if people identify the antecedent in a heuristic way and the probe word 错误 receives the highest level of activation if the resolution is carried out in an exhaustive way.

#### Experiment 2a: The Time Course of Zero Anaphora Resolution Under the Condition of Topic Continuity

##### Subjects

Thirty-two undergraduates with normal or corrected-to-normal vision took part in this experiment. They were all Chinese native speakers.

##### Materials

The experimental materials were composed of 24 sentences, each of which contained 3 clauses, such as“\终于\爬上\了\山顶, Φ眺望\着\远处\高楼林立\的\城市, Φ心想★^1^ 究竟 哪里 才是 自己 的家 啊 ★^2^”The probe words were either true antecedent (e.g., 吴杰) or false antecedent (e.g., 张帆), being inserted either in the position marked ★^1^ or ★^2^. The length of the sentence, the word frequency and character strokes, and the familiarity of the name were properly controlled.

##### Design and Procedure

This experiment was designed as 2 (probe word: true/false antecedent) × 2 (position: ★^1^/★^2^. We examined the period of time consumed by participants in verifying the probe word 250 ms after the characters (e.g., 心想/家啊) were presented on the screen. This experiment was implemented by using E-prime software. In the beginning, “+” was shown on the screen for 600 ms, followed by presenting the materials part by part (each part was identified by “/”). The display time for each part was 300 ms. All the presented parts of the first clause “终于爬上了山顶” disappeared together 300 ms after the last part of this clause “山顶” were given. The next clause followed the same presentation mode. The interval between the disappearance of the verb “心想,” and the occurrence of the probe word was 250 ms. Participants were given 3 s to make judgment whether the word occurred or not. As soon as the decision was made, the rest part continued to appear until the last part disappeared. If participants made wrong judgments, a red sign was presented on the screen. Six hundred millisecond later, they had to answer a question to test their comprehension ability. The wrong answer received feedback. A reading comprehension question was attached to each trial to ensure that participants read carefully. The procedure needed practicing beforehand.

##### Results

We deleted the data that the response time was beyond standard deviations and listed the rest in Table 5. We carried out an analysis of ANOVA on the response time. The results showed that the position had no main effect [*F*_(1, 76)_ = 0.629, *p* > 0.05] but the probe word type produced the main effect [*F*(1,76)=17.402, *p* < .001]. The two variables had the interaction effect [*F*_(1,76)_ = 3.902, *p* = 0.052 (marginal significance)]. Further *t*-test showed that when the probe word was the false antecedent, the position effect was not significant [*t*_(76)_ = 0.525, *p* > 0.05] whereas when the probe word was the true one, the position effect was significant. The response time was faster if the probe word occurred after the sentence than they did after the verb “心想.” Meanwhile, we found that under the condition of topic continuity, the searching for the antecedent did not start until the last character of the essay disappeared. It meant that zero anaphora resolution was possibly carried out in an exhaustive way. We also found that the first-mention effect played a dominant role in zero anaphora resolution.

**Table 5:** Average reaction time to probe words in the position either after the verb or at the end of the sentence under the condition of topic continuity in Experiment 2a (ms).

**Probe word type**	**Probe word location**	
	**After the verb**	**At the end of the sentence**
True antecedent	1157.65	1088.56
False antecedent	1232.11	1252.00

#### Experiment 2b: The Time Course of Zero Anaphora Resolution Under the Condition of Topic Discontinuity

Compared with the situation in which zero anaphora resolution was used when the topic was maintained continuously from sentence to sentence within one essay, the topic discontinuity or new topic insertion prohibited the resolution of Chinese zero anaphora. On one hand, the appearance of more than one antecedent in front of where zero anaphora occurred usually extracted attention. On the other hand, in order to identify the different entities that were mentioned within two or more zero anaphora, people had to consume the increased cognitive efforts. Previous linguistic studies have given a lot of attention to zero anaphora under the condition of topic continuity, we intended to conduct a psycholinguistic experiment to investigate how people resolve zero anaphora when the topic was discontinuous. Since it was one of the possibilities that the antecedent which zero anaphora referred to belonged to either the first mention or the second mention, we preferred to examine whether there was a difference in response time when the probe word was located in different anaphora positions (★^1^/★^2^).

##### Subjects

Another fifty-two undergraduates with normal or correct-to-normal vision took part in the exam.

##### Materials

The experiment materials were composed of 24 sentences with each sentence containing 5 clauses. This was the example: “王锋\突然\听到\一声巨响\, \Φ1迅速\冲出\潜伏的\猫耳洞, Φ1\看到\战友\倒在\前面的\山坡\上, Φ2 \浑身是血\艰难地\挪动★^1^\着, \Φ1便\毫不犹豫地\冲★^2^\了\上去。 \★^3^,” The true antecedents were “王锋” and “战友,” and the false one was “李平.” The “★” stood for the position where the probe word was inserted. We had matched the length of the sentence, the character frequency and strokes, and the familiarity of the names.

##### Design and Procedure

This experiment was designed as 2 (probe word: true/false antecedent) × 3 (position: ★^1^/★^2^/★^3^). We examined the period of time consumed by participants in verifying the probe word 250ms after the characters (e.g., 挪动/冲/上去) were presented on the screen. This experiment was implemented by using E-prime software. The procedure was the same as for Experiment 2a except there were three probe words to be judged on their concurrence after “挪动,” “冲,” and “上去” disappeared respectively.

##### Results

The data beyond 3 standard deviations of the reaction time were deleted (see Table 6). The results of variance analysis of the reaction time between groups were analyzed, showing that the interaction effect was significant [*F*_(2,102)_ = 10.213, *p* < 0.001]. Then, the simple effect analysis between position and probe word type was carried out to investigate the position effect produced both by true antecedent and false antecedent. The results showed that when the antecedent wase true, the position effect was significant with *F*_(2,102)_ = 3.884, *p* < 0.05. The *t*-test further showed that there was a significant difference between ★^1^ and ★^2^ with *t*_(51)_ = −4.553, *p* < 0.001. There was also a significant difference between ★^1^ and ★^3^. The response time was longer when the probe word appeared at the end of the sentence with *t*_(51)_ = −2.321, *p* < 0.05. However, there was no difference between ★^2^ and ★^3^ with *t*_(51)_ = −0.528, *p* > 0.05, indicating that the activation level of probe words in ★^1^ and ★^2^ was higher, so people could access the antecedent much easier.

**Table 6:** Average reaction time to probe words in three positions under the condition of topic discontinuity in Experiment 2b (ms).

**Probe word type**	**Probe word location**		
	**Position 1**	**Position 2**	**Position 3**
True antecedent	1032.00	1171.63	1295.73
False antecedent	1154.83	964.60	1125.65

#### Discussion of the Experiment 2

The results of experiment 2a showed that if the topic was continuous, the activation was at the highest level when the probe word appeared at the end of the sentence. In this situation, zero anaphora resolution was carried out in an exhaustive way. This was consistent with what McDonald and MacWhinney (1995) had proved that the resolution began 250ms rather than immediately after the pronoun appears. Thus, the activation was affected by the position where the probe word was inserted. Meanwhile, the factor of topic continuity was quite important to the anaphoric resolution (e.g., Ariel, 1990, 1994). “When it comes to pronouns, if there is only one entity in the discourse in the focus of attention, processing for pronouns is basically not needed, or the process is automatic” (Greene et al., 1992). The expression “one entity” referred to the topic continuity. It might cause a controversial argument that the topic continuity did not play any significant role in pronoun and zero anaphora resolution in Experiment 1. Why was the case different in Experiment 2? In fact, Experiment 1 and Experiment 2 were designed to explore different aspects of anaphora resolution. Experiment 1 was conducted to require participants to read a sentence and finish a verification judgment after reading. Participants were not interrupted during the time period and they could skip the gap if the topic was not continuous. But the presentation of materials in Experiment 2 was interrupted by the insertion of probe words and the verification task, which forced participants to think about the early information constantly. Therefore, the inconsistent information was very sensitive to them and, therefore, influenced them during the processing. Thus, the topic continuity took different effect. According to Wang and Wu (2020), the change of topic is proven to increase the processing difficulty at some processing stages, but things might be different if anaphora is employed.

Concerning the topic inconsistency, the resolution began when the probe word was present right after the verb. This heuristic way of searching for the antecedent fitted into the structure building model (Gernsbacher, 1990), according to which, the first step to construct comprehension of units larger than a word or image was providing a foundation on which the relations among subsequent events could be laid. Compared with comprehending the topic-maintained discourse, readers had to construct the upcoming information to the current discourse representation and comprehend topic-shifted discourse through building a new substructure for a new topic. People were forced to take different reading strategies in order to achieve a coherent and well-organized representation of the discourse. For Chinese native speakers, as suggested by previous studies, they were used to adopt various reading strategies to cope with the divergent situations: the exhaustive searching strategy for the topic-consistent discourse and the heuristic one for the topic-shifted discourse.

Finally, the insignificant activation of the probe word when they appeared immediately after the verb, which we assumed, did not mean that the resolution was totally inhibited, it was just delayed. According to the investigation of the pronoun anaphora, this tardive resolution was often observed after an interval between the activation of pronoun and the completion of resolution (Sanford and Garrod, 1989; Cristea and Dima, 2001). This was also true to zero anaphora resolution.

### Experiment 3: A Preliminary Study of the Factors That Are Related to the Resolution of Zero Anaphora

Summarizing from previous psycholinguistic experimental studies, we concluded the factors influencing zero anaphora resolution into the aspects such as the topic, the referential distance within one sentence, the verb causality, the voice type (the active/passive voice), and the context where zero anaphora is. We have discussed the first two aspects in the Experiment 1a and 1b. Worth mentioning that, not only the linear distance should be considered as a factor of influencing anaphora resolution, but also the psychological distance, the mental gap which is produced when people are trying to refer back to the antecedent from the position where anaphora appears, should also be taken into account. The linear distance is commonly governed by the grammar rules and each word belongs to a designated position, so that people are not supposed to be influenced by the syntactic structure of linear distance. However, the psychological distance largely varies from people to people in terms of world knowledge and the semantic competence they have. Thus, we are going to discuss the factors related to psychological distance and expected that: (1) if sentences are expressed in an active voice (the first mention priority) in the situation in which the information keeps consistent in meaning within the discourse, the response time to the probe words is the shortest. (2) If the sentences are expressed in the passive voice (non-first mention priority) in the situation in which the information is inconsistent within the discourse, the response time to the probe words is the longest, since there is neither the consistent given information nor the existed first mention priority, people need the largest number of energies to process the new information. Lastly, (3) if the sentences are expressed in the passive voice in the situation in which the information is consistent, or the sentences are expressed in the active voice, but the surrounding information is inconsistent, the reaction time to the probe word is in between.

#### Subjects

The participants were 80 undergraduates with normal or correct-to-normal vision.

#### Materials

The materials were composed of 30 groups of sentences with each group containing 4 different versions of sentences (a total of 120 sentences). Four versions of sentences were exemplified below and were arranged in accordance with the order: (1) the situation described by the first two clauses in the active voice was consistent (the first mentioned priority, e.g., 在课堂上, 老师狠狠批评了班上最调皮的学生, Φ警告他不要再犯错误); (2) the situation described by the first two clauses in the passive voice was consistent (e.g., 在课堂上, 班上最调皮的学生被老师狠狠批评, Φ警告他不要再犯错误); (3) the situation described by the first two clauses in the active voice was inconsistent (the first mentioned priority, e.g., 在医院, 老师狠狠批评了班上最调皮的学生, Φ警告他不要再犯错误); and (4) the situation described by the first two clauses in the passive voice was inconsistent (e.g., 在医院, 班上最调皮的学生被老师狠狠批评, Φ警告他不要再犯错误).

#### Design and Procedure

The experiment was designed as 2 × 2 × 3. The first 2 stood for the voice type (the active voice/the passive voice), the second 2 stood for the situation described by the first two clauses (consistent/inconsistent), and the last 3 stood for the types of probe words (true antecedent with the first mention/true antecedent with the second mention/false antecedent). The dependent variable was the time intervals between the presentation of the probe word and the onset of making a judgment on them. All characters in materials were the most commonly used Chinese characters.

E-prime software was used to implement the experiment. Prior to the experiment, participants were invited to practice three groups of sentences. At the beginning of the experiment, the “+” was displayed on the screen for 600 ms, after that, phrases such as “在课堂上” were presented on the screen character by character, 300 ms for each character. Five hundred milliseconds after the phrase “在课堂上” disappeared, the next part was presented in the same way and stayed on the screen for the same period of time. When the last character of the sentence disappeared, the probe words were shown to participants for 500ms. They were asked to make judgments as accurately and quickly as possible on whether the probe words had been presented or not. If yes, participants pressed the “Y” key, otherwise pressed the “N” key.

#### Results

Table 7 showed the average reaction time to probe words in situations in which the consistent or inconsistent situation was described in active/passive voice in Experiment 3. First of all, we deleted the data of which the response time were beyond standard deviations and conducted ANOVA on the results of response time between groups. The results showed that the interaction effect was not significant [*F*_(1,3)_ = 0.329, *p* > 0.05]. There was a major effect in groups [*F*_(1,3)_ = 3.447, *p* < 0.05], indicating that the response latency of the four groups was significantly different. ANOVA of 2 (voice type: active/passive) × 2 (situation in active voice: consistent/inconsistent) × 2 (probe word type: true antecedent with first-mention/second-mention) was conducted on the experimental data, and the false antecedent was not considered here to further investigate whether there was a difference between active and passive voice and whether there was a significant difference between the consistency and inconsistency of previous information situations.

**Table 7:** Average reaction time to probe words in situations in which the consistent/ inconsistent situation is described in active/ passive voice in Experiment 3 (ms).

**Probe words**	**Active**		**Passive**	
	**consistent**	**inconsistent**	**consistent**	**inconsistent**
True antecedent (first-mention)	573	649	615	687
True antecedent (second-mention)	634	694	657	743
False antecedent	714	749	650	735

It was found that all the interactions were not significant, and the interaction between probe word (true antecedent with first-mention/second-mention) and situation (consistent/inconsistent) was not significant [*F*_(1,79)_ = 0.055, *p* > 0.05]. The interaction between probe word and voice type was not significant [*F*_(1,79)_ = 0.012, *p* > 0.05]. The interaction between voice type and situation was not significant [*F*_(1,79)_ = 0.854, *p* > 0.05]. The interaction among three variables (probe word type, situation, and voice type) was also insignificant [*F*_(1,79)_ = 0.28, *p* > 0.05]. However, the main effect of probe word type was significant [*F*_(1,79)_ = 39.938, *p* < 0.05]. The response of subjects to true antecedent with first mention was faster. Compared with the passive voice, people reacted to sentences with active voice significantly faster with *F*_(1,79)_ = 5.656, *p* < 0.05. The main effect of the information situation was not significant with *F*_(1,79)_ = 2.388, *p* > 0.05.

#### Discussion

What the results have revealed were highly in line with our expectations. If, as the results suggested, the probe words were true antecedents like “老师” (B1) and “学生” (B2), the reaction time under four conditions was systematically different: the shortest reaction time (MB1 = 573 ms, MB2 = 634 ms) and the longest reaction time (MB1 = 687 ms, MB2 = 743 ms) were produced, respectively, when the active voice (the first mention priority) was used in the consistent situation and when the passive voice was used in the inconsistent situation. At the same time, the results under the other two conditions were in between. However, the differences mentioned above were not found when control words were introduced. The effect of voice type was significant, which indicated that the first mention effect (Gernsbacher, 1990) existed under this condition. The effect was also confirmed by Gernsbacher and Hargreaves (1988), in which the advantage of the first mention was found during the processing of the active vs. passive construction in English, despite that the materials did not contain any form of anaphora. It showed that the advantage of first-mention effect is not attributable to the existence of anaphora but the voice type of the language, since it matters that “the foundational role of the earliest participants maintained higher levels of activation than other participants in the same sentence” (MacDonald and MacWhinney, 1990).

The discrepancy in the reaction time was related to the grammatical function the antecedent and zero anaphora played in the whole sentence, i.e., the parallel function. In our experiment, the first type of sentence was processed faster than the second type of sentence, because the antecedent and zero anaphora were the parallel subjects in each clause of the sentence. Similarly, the third type was faster than the fourth type at the processing speed. This was also consistent with Sheldon (1974) and Gernsbacher and Foertsch (1999). Nevertheless, Miao (1996a) found that the parallel function took effect only when the sentence meaning was correct. In another word, if the sentence meaning was correct, the parallel function would not disappear, or vice versa. We cannot examine it very thoroughly, because all materials in our experiment were semantically correct even though the situation they constructed were either consistent or inconsistent. Contrary to our expectations, the effect of situation consistency was insignificant. The first reason, we thought, originated from the failure of the materials in supplying sufficient background information for participants to get ready to refer back to the antecedents and later to resolve the zero anaphora. We carried out the experiment by adopting the research paradigm that Sanford and Garrod (1981) had used, but we revised the words into clauses in our experiment. Theoretically speaking, clauses contained more information than words, but they were not sufficient in assisting participants to identify zero anaphora and refer back to antecedents very well. In other words, the insignificant effect indicated that the resolution of zero anaphora was quite a difficult mental processing. Wang and Yang (2004) admitted that there was a lack of systematic study on the factors related to the underlying psychological mechanism during the pronoun anaphora resolution. This was also true to zero anaphora resolution: the divergent opinions have been formed on the basis of rare but scattered studies and the unsystematic findings of the related factors always vary from one research to another. Compared with pronoun anaphora resolution, zero anaphora resolution encountered more difficulties and required more attention.

## General Discussion

### General Discussion of Experiment Materials

The purpose of the current study is to do the preliminary research to explore the psychological reality of Chinese zero anaphora as a basis for further measuring the time course of resolving sentences containing different types of anaphora, and to make a tentative attempt at determining the factors during this process. The hypotheses we have proposed were partially confirmed: we observed the insignificant effect of the reaction time among all the experimental conditions in Experiment 1, which is consistent with our expectation. The effect of reading time is also insignificant, which is beyond our expectations. The results are possibly due to the indiscriminate activation of probe words without being affected by the anaphora type, that is, zero anaphora resolution is performed as fast as pronoun anaphora resolution. For most Chinese native speakers, the language they speak is characterized by a lot of zero anaphora, which forces the language users to build some corresponding processing models in their minds. Even though it is widely accepted that zero anaphora is an intricate language phenomenon involving mental mechanisms such as short-term memory and psychological distance, Chinese native speakers often successfully overcome the difficulty and refer back to the antecedents very quickly. This is, on another side, reflects how zero anaphora is psychologically realized. What's more, with the finding of the first-mention advantage, the implicit causality effect with subject-biasing than object-biasing verbs is easier to detect (Stewart and Gosselin, 2000). This is consistent with our study. The subject and the first-mentioned entity is processed in privilege because it is mentioned first, so that it is impressed a lot in the minds of people and is easily recognized later.

When we compiled the materials of Experiment 1b, we'd like to insert a changed topic into the material to make the topic discontinuous. The purpose is to distract the attention of participants by introducing another antecedent. Generally speaking, the insertion of a new topic might cause the cognitive competition during the resolution in the brain. When people read the sentence, they usually maintain the first-mention information in their mind, i.e., the information related to the first-mentioned topic and prepare to process it. However, the insertion of a new topic results in the interference of the old information and the mixture of information, including the old information, the new information and the intertwining of them, rushes into the mind so that the complexity of resolution is largely increased. The old information plays a role of constructing a primary concept, which needs constant adjusting in order to coincide with an emerged concept constructed by new information. Therefore, this increased processing procedure delayed the access to the antecedents even they are the true ones.

It is, however, easily challenged by people that the materials we used in our experiments are compiled not as naturally as we read in literature and, at the same time, are lacking of ecological validity since the exclusive adoption of only one narrative style without considering other styles. However, in order to obtain the objective results in psychological empirical studies, we have to narrow down the possibilities by choosing one type of material and deal with them in accordance with the research purpose. Making up the experiment materials is also necessary because the unrelated variables can be controlled as strictly as possible with this method. Although the factitious materials are considered less authentic particularly in terms of expressing meaning, they can meet the requirements that experiments demand in aspects such as word frequency, word length, and syntactic structure. From this perspective, compiling materials can be said critical to the experiment. This is also the way people follow in their experiments.

Experiment 2 investigates the time course of Chinese anaphora resolution. Our expectations partially came true, but under different conditions. When the topic was kept consistent, the searching follows the exhaustive way while when the topic was not kept consistent, the searching follows the heuristic way. We assume that the selection of different searching strategies originates from the capacity of short-term memory. Different from pronoun anaphora resolution, which is obviously signified by the appearance of pronoun, the relationship between the antecedent and its anaphora must be identified through going over the given information back and forth within one sentence or even among sentences. It increases the load of the processing capacity of short-term memory and results in two possibilities: as the topic keeps consistent within the given discourse, the information leads the readers to the end of the discourse without being interrupted by the inserted topic. The exhaustive way of searching for the antecedent does not need such extra energy that short-term memory can fulfill the task. Another possibility is produced when the information inconsistency forces the readers to make sure the anaphora relationship from one place to another until the correct one is identified, thus, most of the time, they use the heuristic way of searching because of the limitation of short-term memory capacity. The insertion of new information breaks down the fluency of reading. Consequently, relatively more cognitive energy is consumed.

The third experiment intends to determine whether the two factors produce the influence on the resolution of zero anaphora on the basis that the resolution has been found psychologically resembled in mind with the way of searching information either heuristically or exhaustively. Out of our expectation, unlike the variable of the voice type, situation consistency does not exert any significant difference on the resolution process. It is not very clear how to interpret the indiscriminate between the consistent and inconsistent situations that the zero anaphora appears. It may be, as we claim, that the materials we used in the experiment fail to supply the sufficient background information in facilitating the participants to form the described situation in mind and identify the referent according to the description. If the situations are not described very distinctively, in other words, if participants are not able to tell the difference between the consistent situation and the inconsistent situation, the insignificant difference is supposed to be observed during the process of making judgments. Concerning the voice type, the active voice and the passive voice, people find it easy to tell the difference just because, according to the Chinese grammar, a defining syntactic indicator “被” is required to occur, which helps people distinguish the voice type even without considering the meaning of the sentences. Consequently, the effect due to the voice type divergence is significantly different, but the situation consistency is not.

Surprisingly enough, by examining the experiment materials further, we discover that when the undergraduates were invited to compile the materials, they preferred to use the verbs like “看 (see),” “发现 (find),” “遇见(meet),” and “留意到(notice)” as the second antecedents. In fact, these verbs are closely related to the vision and sensation of human beings. In Chinese, this is an effective and a common way used to transfer the topic (e.g., 我看见他来了), because Chinese grammar is featured with paradoxical linkage (Song, 2003) with which the syntactic structure is constructed in the relationship of coordination rather than subordination in English. When we compiled the experiment material, we need to consider the language application habits of using synchronic verbs to show the transition from one topic to another in the described situation. Thus, the verbs mentioned above are chosen, which, in turn, has become one of the factors influencing zero anaphora resolution.

With the analysis of materials from another perspective, the position where zero anaphora should have appeared is still a controversial issue. The experiment materials such as type C in Experiment 1a and type B in Experiment 1b were evaluated by the undergraduates majoring in Chinese Literature with Likert scale to confirm whether zero anaphora appears in the right place. For example, in the sentence “中午李明到了学校, 接着Φ (她)开始上课, 课后Φ又到同学那里串门, Φ坐到快天黑时, Φ (她) 才恋恋不舍地告别回家了,” either “Φ” or “她” fits into the place and delivers the same meaning in the position where “Φ (她)” occurs. It shows that zero anaphora and pronoun anaphora can be used alternatively without altering the meaning of the sentence. Gao (2003) investigated the discriminate usage of zero anaphora and pronoun anaphora in written and spoken discourses through pragmatic analysis, finding that the two types of anaphora are used indifferently even the two types of discourse are finished by the same author and in the same literature style. The phenomenon that an anaphoric expression is introduced either by pronoun or zero anaphora brings troubles due to the fact that “return of current discussion to a mention other than the linearly most recent one in the preceding discourse can be done by means of a pronoun or zero anaphora in many languages” (e.g., Huang, 1989, 1994). This is beyond the explanation offered by the recent theory.

In summary, as Greene et al. (1992) argued, the question about pronoun resolution may not be what the pronoun can do for the discourse, but what the discourse can do for the pronoun. As far as zero anaphora resolution is concerned, it is inferred that the question may not be what zero anaphora can do for the discourse, but what the discourse can do for zero anaphora. The anaphoric relationship in discourse is established by nouns, but maintained by pronouns and zero referents (Xiong, 2000).

### General Discussion of the Models

There is a large number of anaphora resolution models, such as Kintsch and van Dijk (1978), Kintsch (1988), and Gernsbacher (1990), but they do not fully account for Chinese zero anaphora resolution. According to the theory of argument overlap (Kintsch, 1988), readers can associate information with previously encoded information by means of argument overlap, which is also considered as an essential way of maintaining referential coherence. However, the theory emphasizes that the overlapping of arguments is the premise of processing information smoothly, or else the lack of it among arguments results in the increased time during the discourse comprehension. The argument is easily found overlapped in the pronoun anaphora for the conspicuous existence of *he, she*, etc. Contrary to that, there are no tangible words in zero anaphora to supply the coherence to people. From this perspective, the argument overlap theory is not able to explain zero anaphora resolution during comprehension. Similarly, the accessibility of the antecedents in sentences containing anaphora expression, particularly the pronoun anaphora expression and the repeated nominal anaphora expression, is proposed to be modulated by the cognitive mechanisms, namely, suppression and enhancement: the presence of the anaphora activating the referents to some degree but prohibiting the non-referents. If, as the typical zero anaphora shows, the anaphora is absent, the underlying mechanism might not take effect during the resolution. Myers and O'Brien (1998) believed that three factors are related to the access to the antecedents in the anaphora expressions, but two of them, that are the special distance between the anaphora and the antecedents as well as the elaborate processing amount of the antecedents, are not clearly determined in resolving the zero anaphora.

The models are proposed to explain the pronoun anaphora resolution in English, but they are not powerful in revealing the underlying mechanism when zero anaphora resolution is used in Chinese. Language is a means of communication and is expected to be used to transfer information as accurately and efficiently as possible. Zero anaphora is commonly seen in Chinese and is welcomed by people for its efficiency and accuracy in delivering information. Language reflects the way of thinking and, in turn, is significantly affected by it, so people who speak Chinese have been used to thinking in the way that the language determines and have developed the strategies to deal with the expressions containing zero anaphora. This was proven by Tao and Healy (2005) in their experiment, in which Chinese native speakers were found to show the advantage over English native speakers in coping with zero anaphora even when the materials were presented in English, indicating that Chinese native speakers have transferred such a strategy from one language to another.

What is more, compared with ideal zero anaphora in our experiment, the practical use of zero anaphora is quite complicated. Zero anaphora is just a composition of the “anaphora chain,” reflecting the partial rather than the overall meaning that the discourse intends to express. Like the hoops buckling into a chain, each anaphora contains one idea, and the scattering ideas are organized by the chain to produce a comprehensive one and form the coherence of the discourse at the same time. Li and Thompson (1979) use “topic chain” to describe this language phenomenon, pointing out that the topic established in the first clause serves as the referent for the unrealized topics in the following clauses. Tsao (1990) claims that “a sentence in Chinese can be roughly defined as a topic chain, which, is a stretch of discourse composed of one or more comment clauses sharing a common topic, which heads the chain.” Chen (1987) claims that zero anaphora or pronoun anaphora is responsible for encoding the referents that possess high topic continuity in Chinese. However, the question still exists, namely, to what extent and in what aspects the topic chain facilitates the encoding of zero anaphora resolution.

In general, anaphora resolution has received the close attention from disciplines such as psycholinguistics, Chinese linguistics, computational linguistics, pragmatics, and philosophy. As we have mentioned, zero anaphora is more commonly seen in Chinese rather than in Indo-European languages. Studying zero anaphora resolution in depth can enrich our linguistic knowledge about Chinese and supply enlightenment to understand the philosophy among languages and the way of thinking and cultures. At the same time, it helps solve some key problems in machine translation and computer processing of natural language.

It is important to remember the limitations of the current study. First, we intended to test our hypothesis by employing the real-time processing methods, but as the results suggested, not all the hypotheses have been empirically tested. It is for sure that the real-time measurement is a way to reveal the underlying mechanism above which zero anaphora resolution is performed, but it is unable to tell the whole story of reading comprehension. For example, priming and detection technology are specialized at activating, representing, and organizing information, but may disrupt the reading fluency (Tanenhaus, 2004). Considering that anaphora resolution occurs immediately after the trigger is given, the results are less reliable if the detection words are introduced between the antecedents and the zero anaphora. Thus, if possible, more on-line research methods such as ERPs and fMRI should be considered as an alternative way to find out more subtle differences that are not significantly resembled in the present study. Second, the insignificant differences may be due to the design of the experiment. Specifically, although zero anaphora resolution depends on grammatical and syntactical knowledge, it requires background knowledge at the same time. For example, the two sentences “老张生了个儿子, Φ天天哭闹,” and “老张生了个儿子, Φ天天炫耀” are constructed identically in grammar and syntactic structure, the antecedents that zero anaphora refers back are quite different with “儿子” in the first sentence and “老张” in the second. It is conceivable that people with background knowledge may indeed exert a great influence on the resolution of zero anaphora in very different ways. Third, further thinking might concern but might not be restricted to the following aspects: (a) The pictographic form of Chinese characters possibly contains more “information load” than Latin words, which is assumed to be highly related to identify zero anaphora and facilitate its resolution. (b) Chinese native speakers usually think in a holistic and systematic way, which potentially cultivates their ability of grasping the main idea even if the sentences are incomplete in grammar. As for zero anaphora, the lack of referent in certain places does not stop them from comprehending the meaning of the sentence. According to Dopkins et al. (1992), anaphora resolution, in most cases, is either a bottom-up or top-down procedure, we assume that Chinese native speakers tend to construct a situational model in discourse comprehension. As a result, zero anaphora resolution is a top-down procedure. (c) The adding of some Chinese adverbs in experiment materials such as “也 (also)” “只好 (have to)” in the sentences improves the logical relationship among clauses where zero anaphora is contained. We just wonder whether the fluent resolution of zero anaphora is also determined using adverbs. If so, in what way does the use of adverbs affect the resolution.

## Conclusion

Initiated by the idea that Chinese zero anaphora resolution might involve a unique way of processing information that is quite different from the way of processing other types of anaphora, we carried out five experiments to test three hypotheses from the perspectives of the psychological reality of the resolution, the time courses of the resolution and the factors related to the resolution. Based on the results, we conclude that the first-mention effect exists during zero anaphora resolution, which follows the same processing mechanism with pronoun resolution. During the resolution, the exhaustive way of searching for the antecedents within one sentence happens when the topic is in consistency, but the heuristic way of searching is carried out when the topic is out of consistency. Meanwhile, the first-mention effect is also found when the discourse is organized in the active voice no matter the information within the discourse is consistent or not.

## Data Availability Statement

The original contributions presented in the study are included in the article/supplementary material, further inquiries can be directed to the corresponding author/s.

## Ethics Statement

The studies involving human participants were reviewed and approved by Review Board of South China Normal University. The patients/participants provided their written informed consent to participate in this study.

## Author Contributions

NY and ZL designed the study. NY, JZ, and LM acquired the data. NY and LM analyzed the data. JZ wrote the manuscript. ZL polished it. All authors contributed equally to reviewing the manuscript.

## Funding

This work was supported by the Key Program of National Fund of Philosophy and Social Science of China (19AYY014), Major Program of Guangdong Provincial Fund of Philosophy and Social Sciences (GD20ZDWY02), Bidding Project of 2021 of the Key Lab for Social Sciences of Guangdong Province, Bilingual Cognition and Development Lab (BCD202103), Project of 13th Five-Year Plan of Philosophy and Social Science Development in Guangzhou (2019GZYB55 and 2020GZGJ277), Project of 13th Five-Year Plan of Philosophy and Social Science Development in Guangdong (GD20XWY05 and GD20XXL01). Major Cultivating Project of Guangdong University of Foreign Studies (20ZD01); Distinguished Innovation Project of Guangdong University of Foreign Studies (19SS03).

## Conflict of Interest

The authors declare that the research was conducted in the absence of any commercial or financial relationships that could be construed as a potential conflict of interest.

## Publisher's Note

All claims expressed in this article are solely those of the authors and do not necessarily represent those of their affiliated organizations, or those of the publisher, the editors and the reviewers. Any product that may be evaluated in this article, or claim that may be made by its manufacturer, is not guaranteed or endorsed by the publisher.

## Footnotes

^1^The five-sentence essays are constructed on the basis of Chen (1987) for the reason that the structure containing anaphora objects and the antecedents in discourse are generally not more than three levels, that is, not more than five sentences.
