# Supplementary material for: A Study of Zero Anaphora Resolution in Chinese Discourse: From the Perspective of Psycholinguistics
Source: Front Psychol. 2021 Oct 28;12:663168. doi: 10.3389/fpsyg.2021.663168 (PMC8581763; doi:10.3389/fpsyg.2021.663168)
Supplement: Supplementary file 1 [file Data_Sheet_1.pdf]

Appendix

Materials of Experiment 1a

1a. 中午李明到了学校\，接着她开始上课\，课后她又到同学那里串门\，她坐到快天黑时\，她才恋恋不舍地告别回家了\。

making judgment：李明课后去拜访同学（T）

1b. 中午李明到了学校\，接着开始上课\，课后又到同学那里串门\，坐到快天黑时\，才恋恋不舍地告别回家了\。

making judgment：李明课后直接回家了 （F）

1c. 中午李明到了学校\，接着她开始上课\，课后又到同学那里串门\，坐到快天黑时，她才恋恋不舍地告别回家了\。

making judgment：李明课后没有直接回家（T）

probe word: 李明（T）林叶（F）

2a. 周末张卫起得很迟\，他吃了一顿丰盛的早餐\，他突然想起有客人要来\，于是他把房子打扫干净\，然后他决定去超市买菜\。

making judgment：张卫很晚才去买菜（T）

2b. 周末张卫起得很迟\，吃了一顿丰盛的早餐\，突然想起有客人要来\，于是把房子打扫干净\，然后决定去超市买菜\。

making judgment：张卫一早就去买菜（F）

2c. 周末张卫起得很迟\，他吃了一顿丰盛的早餐\，突然想起有客人要来\，于是把房子打扫干净\，然后他决定去超市买菜\。

making judgment：张卫根本就没有买菜（T）

probe word: 张卫（T）刘洪（F）

3a. 清晨赵伟准备骑车出门\，他出门走到自行车棚\，他发现车胎已经没气了\，他拿出气筒给车打气\，他不小心把车胎打爆了\。

making judgment：赵伟不小心把车胎打爆了（T）

3b. 清晨赵伟准备骑车出门\，出门走到自行车棚\，发现车胎已经没气了\，拿出气筒给车打气\，不小心把车胎打爆了\。

making judgment：赵伟故意把车胎打爆了（F）

3c. 清晨赵伟准备骑车出门\，他出门走到自行车棚\，发现车胎已经没气了\，他拿出气筒给车打气\，不小心把车胎打爆了\。

making judgment：赵伟故意把车胎打爆了（F）

probe word：赵伟（T）郑彪（F）

4a. 天气热得李芒有点受不了\，整个下午他都热得无法看书\，他打算装台空调\，他在商场逛了半天\，最后他下决心购买格力空调\。

making judgment：李芒决定购买格力空调（T）

4b. 天气热得李芒有点受不了\，整个下午都热得无法看书\，打算装台空调\，在商场逛了半天\，最后下决心购买格力空调\。

making judgment：李芒不知道买什么空调（F）

4c. 天气热得李芒有点受不了\，整个下午都热得无法看书\，他打算装台空调\，他在商场逛了半天\，最后下决心购买格力空调\。

making judgment：李芒不打算买任何空调（F）

probe word：李芒（T）张川（F）

5a. 早晨王强来到长途汽车站\，无奈他错过了早班车\，他只好买张中午的车票\，他在候车室里等得百无聊赖\，他只好看着车站里人来人往\。

making judgment：王强在车站等下一班车（T）

5b. 早晨王强来到长途汽车站\，无奈错过了早班车\，只好买张中午的车票\，在候车室里等得百无聊赖\，只好看着车站里人来人往\。

making judgment：王强错过了一班车（T）

5c. 早晨王强来到长途汽车站\，无奈错过了早班车\，他只好买张中午的车票\，在候车室里等得百无聊赖\，他只好看着车站里人来人往\。

making judgment：王强在车站打扫卫生（F）

probe word：王强（T）陈伟（F）

6a. 林冰边吃饭边看电视\，她看到关于海南的景点介绍\，她觉得去海南旅游不错\，她仔细翻阅今天的报纸\，她终于找到一家中意的旅行社\。

making judgment：林冰有意去海南旅游（T）

6b. 林冰边吃饭边看电视\，看到关于海南的景点介绍\，觉得去海南旅游不错\，仔细翻阅今天的报纸\，终于找到一家中意的旅行社\。

making judgment：林冰只是看看海南的介绍（F）

6c. 林冰边吃饭边看电视\，她看到关于海南的景点介绍\，觉得去海南旅游不错\，她仔细翻阅今天的报纸\，终于找到一家中意的旅行社\。

making judgment：林冰只是想了解一下海南（F）

probe word：林冰（T）沈丽（F）

7a. 刘明决定晚上去看电影\，他匆匆骑车来到电影院\，他发现电影已经开演\，他只好买了晚一场的票\，他坐在大门台阶上无聊地消磨时间\。

making judgment：刘明正在等下一场电影（T）

7b. 刘明决定晚上去看电影\，匆匆骑车来到电影院\，发现电影已经开演\，只好买了晚一场的票\，坐在大门台阶上无聊地消磨时间\。

making judgment：刘明刚刚看了一场电影（F）

7c. 刘明决定晚上去看电影\，他匆匆骑车来到电影院\，发现电影已经开演\，他只好买了晚一场的票\，坐在大门台阶上无聊地消磨时间\。

making judgment：刘明刚刚错过一场电影（T）

probe word：刘明（T）李辉（F）

8a. 李平今天参加职称考试\，他满怀信心步入考场\，他把有关证件放在桌面上\，他耐心等待着考试开始\，突然他感到肚子一阵难受\。

making judgment：李平感到肚子不大好受（T）

8b. 李平今天参加职称考试\，满怀信心步入考场\，把有关证件放在桌面上\，耐心等待着考试开始\，突然感到肚子一阵难受\。

making judgment：李平感到心情不大好受（F）

8c. 李平今天参加职称考试\，他满怀信心步入考场\，把有关证件放在桌面上\，耐心等待着考试开始\，突然他感到肚子一阵难受\。

making judgment：李平感到信心不太充足（F）

probe word：李平（T）吴伟（F）

9a. 王成决定晚上去看电影\, 他匆匆来到电影院\, 他刚好碰到以前的老同事\, 他连忙买了两张票\, 他说要请客看电影\。

making judgment：王成说要请客看电影（T）

9b. 王成决定晚上去看电影\, 匆匆来到电影院\, 刚好碰到以前的老同事\, 连忙买了两张票\, 说要请客看电影\。

making judgment：王成说要独自看电影（F）

9c. 王成决定晚上去看电影\, 他匆匆来到电影院\, 刚好碰到以前的老同事\, 他连忙买了两张票\, 说要请客看电影\。

making judgment：王成不太喜欢看电影（F）

probe word：王成（T）黄静（F）

10a. 李剑走进路边的杂货点\, 他想买瓶汽水解解渴\, 他掏出钱交给店员\, 然后他接过冰凉的汽水\, 他很快就喝完了\。

making judgment：李剑觉得嗓子快要冒烟（T）

10b. 李剑走进路边的杂货点\, 想买瓶汽水解解渴\, 掏出钱交给店员\, 然后接过冰凉的汽水\, 很快就喝完了\。

making judgment：李剑用水把烟浇灭了（F）

10c. 李剑走进路边的杂货点\, 想买瓶汽水解解渴\, 他掏出钱交给店员\, 然后接过冰凉的汽水\, 很快就喝完了\。

making judgment：李剑一口气把水喝完了（F）

probe word：李剑（T）孙武（F）

11a. 朱军打算去练口语\, 他一早就来到英语角\, 他跟一个外教聊了几句\, 他觉得总是词不达意\, 他痛感自己的口语不好\。

making judgment：朱军很想提高口语水平（T）

11b. 朱军打算去练口语\, 一早就来到英语角\, 跟一个外教聊了几句\, 觉得总是词不达意\, 痛感自己的口语不好\。

making judgment：朱军特别喜欢表现自己（F）

11c. 朱军打算去练口语\, 他一早就来到英语角\, 跟一个外教聊了几句\, 觉得总是词不达意\, 他痛感自己的口语不好\。

making judgment：朱军特别痛恨自己（F）

probe word：朱军（T）王宁（F）

12a. 张灵走进自习室\，她找了个靠窗的位置\，她顺手帮同学占了个座位\，她拿出一本英语考研试题书\，她仔细阅读英语写作范文\。

making judgment：张灵正在自习室读书（T）

12b. 张灵走进自习室\，找了个靠窗的位置\，顺手帮同学占了个座位\，拿出一本英语考研试题书\，仔细阅读英语写作范文\。

making judgment：张灵一点不喜欢英语（F）

12c. 张灵走进自习室\，她找了个靠窗的位置\，顺手帮同学占了个座位\，她拿出一本英语考研试题书\，仔细阅读英语写作范文\。

making judgment：张灵正在帮人占座位（T）

probe word：张灵（T）陈慧（F）

13a. 一下班徐刚就冲出办公室\，他很快就来到附近的咖啡馆\，他约了朋友一起吃晚饭\，他先要了一杯开水\，然后他拿出报纸翻看\。

making judgment：徐刚要和别人一起吃晚饭（T）

13b. 一下班徐刚就冲出办公室\，很快就来到附近的咖啡馆\，约了朋友一起吃晚饭\，先要了一杯开水\，然后拿出报纸翻看\。

making judgment：徐刚下班后回家吃晚饭（F）

13c. 一下班徐刚就冲出办公室\，很快就来到附近的咖啡馆\，他约了朋友一起吃晚饭\，他先要了一杯开水\，然后拿出报纸翻看\。

making judgment：徐刚下班后直接去咖啡馆（T）

probe word：徐刚（T）贺兵（F）

14a. 王锋听到一声巨响\，他迅速冲出猫耳洞\，他看到战友倒在前面的山坡上\，突然他感到大腿一阵发麻\，原来他中了敌人的冷枪\。

making judgment：王锋的大腿被打伤了（T）

14b. 王锋听到一声巨响\，迅速冲出猫耳洞\，看到战友倒在前面的山坡上\，突然感到大腿一阵发麻\，原来中了敌人的冷枪\。

making judgment：王锋的腿被蚊子叮了（F）

14c. 王锋听到一声巨响\，他迅速冲出猫耳洞\，看到战友倒在前面的山坡上\，突然感到大腿一阵发麻\，原来他中了敌人的冷枪\。

making judgment：王锋看到战友受伤了（T）

probe word：王锋（T）张岩（F）

15a. 孙琳周末去逛街\，她坐车来到北京路\，她一家商店挨一家商店地逛\，她觉得又累又饿\，于是她决定找个地方吃饭\。

making judgment：孙琳买了许多东西（T）

15b. 孙琳周末去逛街\，坐车来到北京路\，一家商店挨一家商店地逛\，觉得又累又饿\，于是决定找个地方吃饭\。

making judgment：孙琳吃了许多东西（F）

15c. 孙琳周末去逛街\，她坐车来到北京路\，一家商店挨一家商店地逛\，她觉得又累又饿\，于是决定找个地方吃饭\。

making judgment：孙琳逛了许多商店（T）

probe word：孙琳（T）王玲（F）

16a. 林红掏出房门钥匙\，她轻轻地打开门\，她打开大厅的灯\，她懒洋洋地躺在大厅的沙发上\，她觉得自己的骨头都快散架了\。

making judgment：林红觉得非常疲倦（T）

16b. 林红掏出房门钥匙\，轻轻地打开门\，打开大厅的灯\，懒洋洋地躺在大厅的沙发上\，觉得自己的骨头都快散架了\。

making judgment：林红的骨头快断了（F）

16c. 林红掏出房门钥匙\，轻轻地打开门\，打开大厅的灯\，她懒洋洋地躺在大厅的沙发上\，觉得自己的骨头都快散架了\。

making judgment：林红是个很懒的人（F）

probe word：林红（T）夏青（F）

17a.下午吴岩坐在窗前看书\，突然他发现外面下雨了\，他连忙冲到阳台上\，他开始收拾晒在外面的衣物\，他发现衣服已经差不多全湿了\。

making judgment：吴岩发现衣服全湿了（T）

17b.下午吴岩坐在窗前看书\，突然发现外面下雨了\，连忙冲到阳台上\，开始收拾晒在外面的衣物\，发现衣服已经差不多全湿了\。

making judgment：吴岩匆匆忙忙收衣服（T）

17c. 下午吴岩坐在窗前看书\，突然发现外面下雨了\，他连忙冲到阳台上\，开始收拾晒在外面的衣物\，他发现衣服已经差不多全湿了\。

making judgment：吴岩发现衣服还是干的（F）

probe word：吴岩（T）赵刚（F）

18a.天刚亮王钢就起了床\，他发现时间其实不早了\，他顾不上洗脸刷牙\，他收拾好书包\，他飞快地冲到公共汽车站\。

making judgment：王钢特别担心自己迟到（T）

18b.天刚亮王钢就起了床\，发现时间其实不早了\，顾不上洗脸刷牙\，收拾好书包\，飞快地冲到公共汽车站\。

making judgment：王钢特别喜欢早晨长跑（F）

18c. 天刚亮王钢就起了床\，他发现时间其实不早了\，顾不上洗脸刷牙\，收拾好书包\，飞快地冲到公共汽车站\。

making judgment：王钢发现时间已经不早了（T）

probe word：王钢（T）李平（F）

19a.晚饭后邓义准备出去走走\，他刚刚走出家门\，他听到客厅的电话铃响了\，他赶忙返回去\，他听到一个熟悉的声音\。

making judgment：邓义的电话是朋友打来的（T）

19b.晚饭后邓义准备出去走走\，刚刚走出家门\，听到客厅的电话铃响了\，赶忙返回去\，听到一个熟悉的声音\。

making judgment：邓义的电话是陌生人打来的（F）

19c. 晚饭后邓义准备出去走走\，他刚刚走出家门\，听到客厅的电话铃响了\，他赶忙返回去\，听到一个熟悉的声音\。

making judgment：邓义的电话是家里人打来的（F）

probe word：邓义（T）吴英（F）

Materials of Experiment 1b

1a. 一大早醒来王锋突然听到一声巨响\，迅速冲出潜伏的猫耳洞\，看到战友倒在前面的山坡上\，浑身是血艰难地挪动着\，便毫不犹豫地冲了上去。

making judgment：王锋舍身抢救战友（T）

1b. 一大早醒来王锋突然听到一声巨响\，他迅速冲出潜伏的猫耳洞\，看到战友倒在前面的山坡上\，浑身是血艰难地挪动着\，便毫不犹豫地冲了上去。

making judgment：战友舍身抢救王锋（F）

probe word：王锋（T）战友（T）李平（F）

2a. 林冰放学后去喂鸭子\，把鸭子赶到村旁的池塘里\，突然看见几个小孩赤身露体\，一起在池塘追逐打闹\，大声叫他们要小心。

making judgment：林冰跟人在池塘里打闹（F）

2b. 林冰放学后去喂鸭子\，她把鸭子赶到村旁的池塘里\，突然看见几个小孩赤身露体\，一起在池塘追逐打闹\，她大声叫他们要小心。

making judgment：小孩们正在池塘玩耍（T）

probe word：林冰（T）小孩（T）杨艺（F）

3a. 晚饭后邓义准备出去走走\，刚刚走出家门\，就遇到前来探访的乡下亲戚\，亲戚拎着一个鼓鼓囊囊的袋子\，心想又有什么事了。

making judgment：邓义的亲戚又来拜访了（T）

3b. 晚饭后邓义准备出去走走\，刚刚走出家门\，就遇到前来探访的乡下亲戚\，拎着一个鼓鼓囊囊的袋子\，邓义心想又有什么事了。

making judgment：晚饭后邓义出门去接亲戚（F）

probe word：邓义（T）亲戚（T）张灵（F）

4a.下午李明正在图书馆安静地读书\，突然听见身边有声音\，仔细一看原来有人鬼鬼祟祟四处张望\，个子不高蒙着头套\，不禁想这人到底要干什么。

making judgment：李明正在到处张望（F）

4b.下午李明正在图书馆安静地读书\，突然听见身边有声音\，她仔细一看原来有人鬼鬼祟祟四处张望\，个子不高蒙着头套\，李明不禁想这人到底要干什么。

making judgment：李明在图书馆读书（T）

probe word：李明（T）有人（T）刘伟（F）

5a.张莉周末去公园游玩\，见到一个熟悉的身影\，发现原来是很久不见的朋友\，长得完全都变了模样\，赶忙上前去打招呼。

making judgment：张莉遇见了多年不见的朋友（T）

5b.张莉周末去公园游玩\，她见到一个熟悉的身影\，发现原来是很久不见的朋友\，长得完全都变了模样\，她赶忙上前去打招呼。

making judgment：几年不见张莉完全都变了模样（F）

probe word：张丽（T）朋友（T）钱宁（F）

6a.李平今天参加职称考试\，满怀信心步入考场\，突然听到有人喝令他站住\，气势汹汹地叫他拿出证件\，觉得非常反感\。

making judgment：李平气势汹汹地拿出证件（F）

6b. 李平今天参加职称考试\，他满怀信心步入考场\，突然听到有人喝令他站住\，气势汹汹地叫他拿出证件\，他觉得非常反感\。

making judgment：李平对监考人的态度非常不满（T）

probe word：李平（T）有人（T）张宏（F）

7a. 杨辉晚饭后到湖边散步\，突然听到有人喊"救命"的声音\，见是一个小孩掉进了湖里\，正在痛苦地挣扎着\，便二话不说马上跳进湖中\。

making judgment：杨辉毫不犹豫跳进湖中救人（T）

7 b. 杨辉晚饭后到湖边散步\，突然听到有人喊"救命"的声音\，见是一个小孩掉进了湖里\，正在痛苦地挣扎着\，他便二话不说马上跳进湖中\。

making judgment：杨辉在湖中痛苦地挣扎（F）

probe word：杨辉（T）小孩（T）王玲（F）

8a. 中午陈娜走在放学回家的路上\，刚刚准备过马路\，看见一个老奶奶拿着很多东西\，步履蹒跚地也要过马路\，赶忙上前去帮忙\。

making judgment：陈娜特别喜欢逛马路（F）

8b. 中午陈娜走在放学回家的路上\，她刚刚准备过马路\，看见一个老奶奶拿着很多东西\，步履蹒跚地也

要过马路\，陈娜赶忙上前去帮忙\。

making judgment：陈娜是一个乐于助人的学生（T）

probe word：陈娜（T）老奶奶（T）张华（F）

9a. 王萍周末到书店买书\，高高兴兴来到购书中心\，看见很多小学生\，穿着校服戴着红领巾\，心想今天莫非有什么活动\。

making judgment：王萍穿着校服戴着红领巾（F）

9b. 王萍周末到书店买书\，她高高兴兴来到购书中心\，看见很多小学生\，穿着校服戴着红领巾\，王萍心想今天莫非有什么活动\。

making judgment：王萍到购书中心买书（T）

probe word：王平（T）小学生（T）朱军（F）

10a. 一大早清张成正在静静地睡觉\，突然听到急促的敲门声\，开门一看是一位很久不见的叔叔\，满头大汗神色慌张\，忙问出了什么事情\。

making judgment：张成想知道出了什么事情（T）

10b. 一大早张成正在静静地睡觉\，突然听到急促的敲门声\，开门一看是一位很久不见的叔叔\，满头大汗神色慌张\，张成忙问出了什么事情\。

making judgment：张成知道发生了什么事情（F）

probe word：张成（T）叔叔（T）王永（F）

11a. 下午五点多张宇刚刚走到小区门口\，突然听到咚的一声\，发现不远处一个小孩从地上爬起来\，正用力拍打着书包\，心想肯定是小孩摔跟头了\。

making judgment：小孩在小区门口摔了一个跟头（T）

11b.下午五点多张宇刚刚走到小区门口\，突然听到咚的一声\，发现不远处一个小孩从地上爬起来\，正用力拍打着书包\，张宇心想肯定是小孩摔跟头了\。

making judgment：张宇在小区门口摔了一个跟头（F）

probe word：张宇（T）小孩（T）叶平（F）

12a. 李红晚上在家里做作业\，忽然听见楼下草地上有声音\，原来是邻居在练武\，把一套拳打得如行云流水一般\，不禁暗暗叫好\。

making judgment：李红特别喜欢练武（F）

12b. 李红晚上在家里做作业\，忽然听见楼下草地上有声音\，原来是邻居在练武\，把一套拳打得如行云流水一般\，李红不禁暗暗叫好\。

making judgment：李红暗暗夸邻居的武艺（T）

probe word：李红（T）邻居（T）王永（F）

13a. 黄昏时分方明走路去表哥家里\，过了一会发现自己迷路了\，好不容易见到一位警察\，非常热心地指路\，心里不禁涌上感激之情\。

making judgment：方明很感激表哥（F）

13b. 黄昏时分方明走路去表哥家里\，过了一会他发现自己迷路了\，好不容易见到一位警察\，非常热心地指路\，他心里不禁涌上感激之情\。

making judgment：方明很感激警察（T）

probe word：方明（T）警察（T）高兰（F）

14a. 一天张军正在校园里画着写生\，耳边传来银铃般的笑声\，原来是一群少女在开心地玩耍\，长得都非常活泼可爱\，不禁想把这一幕记录下来\。

making judgment：张军正在校园里写生（T）

14b. 一天张军正在校园里画着写生\，耳边传来银铃般的笑声\，原来是一群少女在开心地玩耍\，长得都非常活泼可爱\，他不禁想把这一幕记录下来\。

making judgment：张军正在开心地玩耍（F）

probe word：张军（T）少女（T）孙伟（F）

15a. 今天李宁第一次参加跆拳道比赛\，忐忑不安来到比赛场地\，见到了自己的对手\，长得高高大大\，心里感到十分紧张\。

making judgment：李宁有些害怕对手（T）

15b. 今天李宁第一次参加跆拳道比赛\，他忐忑不安来到比赛场地\，见到了自己的对手\，长得高高大大\，他心里感到十分紧张\。

making judgment：李宁在精神上压倒对手（F）

probe word：李宁 （T）对手 （T）徐征（F）

16a. 新学期刘芳终于转到了新的学校，一大早便高兴地来到学校，见到了新的老师，非常和蔼可亲，打心眼里觉得喜欢\。

making judgment：刘芳转到了新的学校（T）

16b. 新学期刘芳终于转到了新的学校，她一大早便高兴地来到学校，见到了新的老师，非常和蔼可亲，刘芳打心眼里觉得喜欢。

making judgment：刘芳对新学校没有什么印象（F）

probe word：刘芳（T）老师（T）王双（F）

17a. 昨天赵华参加同事的生日晚会\，见到了很多熟人\，注意到一位很特别的女生\，穿着朴素的校服\，总觉得有几分眼熟\。

making judgment：赵华在晚会上见到了一个熟人（F）

17b. 昨天赵华参加同事的生日晚会\，见到了很多熟人\，他注意到一位很特别的女生\，穿着朴素的校服\，她总觉得有几分眼熟\。

making judgment：赵华在晚会上见到了很多熟人（T）

probe word：赵华（T）女生（T）金立（F）

18a. 傍晚王红坐车去看演唱会\，高兴地来到体育场\，终于见到自己心仪已久的歌星\，长得青春靓丽英俊潇洒\，高兴得忘记自己姓什么了\。

making judgment：王红长得青春靓丽（F）

18b. 傍晚王红坐车去看演唱会\，她高高兴兴地来到体育场\，终于见到自己心仪已久的歌星\，歌星长得青春靓丽英俊潇洒\，王红高兴得忘记自己姓什么了\。

making judgment：歌星长得青春靓丽（T）

probe word：王红（T）歌星（T）刘青（F）

19 a. 不一会陈立放下电话\，匆匆赶往校门口\，远远看见母亲就站在校门旁\，正跟门卫比划着什么\，不由得赶快冲了过去\。

making judgment：陈立在校门口见到了母亲（T）

19 b. 不一会陈立放下电话\，匆匆赶往校门口\，远远看见母亲就站在校门旁\，正跟门卫比划着什么\，他不由得赶快冲了过去\。

making judgment：陈立与门卫发生了冲突（F）

probe word：陈立（T）母亲（T）刘芳（F）

20a. 清晨刘刚正要过马路\，突然看见有一个身影走过来\，发现是一位警察\，不停地挥手示意\，于是赶紧停住脚步\。

making judgment：刘刚受到警察的批评（F）

20b. 清晨刘刚正要过马路\，突然他看见有一个身影走过来\，发现是一位警察\，不停地挥手示意\，于是他赶紧停住脚步\。

making judgment：刘刚受到警察的表扬（F）

probe word：刘刚（T ）警察（T ）王炎（F）

21a. 黄丽早上在等校车\，见到马路边有人打招呼\，认出是同学陈琳\，抱怨说今天校车坏了\，心想今天要打的士去学校了\。

making judgment：吴霞想打的士去学校（F）

21b. 黄丽早上在等校车\，见到马路边有人打招呼\，她认出是同学陈琳\，抱怨说今天校车坏了\，黄丽心想今天要打的士去学校了\。

making judgment：陈琳想打的士去学校（T）

probe word：黄丽（T ）陈琳（T ）丁燕（F）

22a. 张红上午去菜市场买菜\，在市场门口看见许多人围成一堆\，走近后发现是一个乞丐躺在地上\，正用粉笔写着什么\，禁不住暗暗好奇\。

making judgment：张红给了乞丐一点钱（F）

22b. 张红上午去菜市场买菜\，在市场门口看见许多人围成一堆\，走近后发现是一个乞丐躺在地上\，正用粉笔写着什么\，她禁不住暗暗好奇\。

making judgment：张红对乞丐的行为感到好奇（T）

probe word：张红（T）乞丐（T）吴非（F）

23 a. 李勇坐在公车上昏昏欲睡\，隐约感到提包不见了\，抬头一看有人正把手缩回去\，并装着若无其事的样子\，于是用力一拽把包夺了回来\。

making judgment：李勇的包差点被偷了（T）

23 b. 李勇坐在公车上昏昏欲睡\，隐约感到提包不见了\，他抬头一看有人正把手缩回去\，并装着若无其

事的样子\，于是他用力一拽把包夺了回来\。

making judgment：李勇装着若无其事的样子（F）

probe word：刘刚（T）有人（T）李锐（F）

24 a. 下午王娟正照顾生病的妈妈\，突然听到敲门的声音\，见是隔壁的阿姨\，拿着一篮新鲜的水果\，忙把她请进屋里\。

making judgment：王娟听到敲门的声音（T）

24 b. 下午王娟正照顾生病的妈妈\，突然她听到敲门的声音\，见是隔壁的阿姨\，拿着一篮新鲜的水果\，王娟忙把她请进屋里\。

making judgment：妈妈听到敲门的声音（F）

probe word：王娟（T）阿姨（T）周燕（F）

Materials of Experiment 2a

1. 李明\到\同学\家里\串门\，坐到\快\天黑\时\，才\恋恋不舍\地\告别\回家了\。

probe word：李明（T）林叶（F）

making judgment：李明在同学家坐了很久（T）

2. 今天\张卫\起得\很晚\，吃\了\一顿\丰盛\的\早餐\，突然\想起\还有\客人\要来\。

probe word：张卫（T）刘洪（F）

making judgment：张卫想起早餐还没有吃（F）

3. 清晨\赵伟\准备\骑车\出门\，出门\走到\自行车棚\，发现\车胎\已经\没气了\。

probe word：赵伟（T）郑彪（F）

making judgment：赵伟发现自行车被偷了（F）

4. 天气\热得\李芒\有点\受不了\，整个\下午\都\看\不进\书\，心想\还是\装\台\空调\吧\。

probe word：李芒（T）张川（F）

making judgment：李芒热得看不下书（T）

5. 早晨\王强\来\到\长途汽车站\，无奈\错过了\早班车\，只好买\张\中午的\车票\。

probe word：王强（T）陈伟（F）

making judgment：王强顺利坐上了长途汽车（F）

6. 林冰\边吃\饭\边看\电视\，看到\关于\海南\的\景点\介绍\，觉得\去\海南\旅游\不错\。

probe word：林冰（T）沈娜（F）

making judgment：林冰觉得去海南旅游是个不错的选择（T）

7. 刘明\决定\晚上\去看\电影\，匆匆\骑车\来到\电影院\，发现\电影\已经\开演\。

probe word：刘明（T）李辉（F）

making judgment：刘明开车去看电影（F）

8. 晚饭\后\邓义\准备\出去\走走\，刚\走出\家门\，便\听到\客厅\的\电话铃\响了\。

probe word：邓义（T）吴伟（F）

making judgment：邓义一出门家里的电话就响了（T）

9. 王成\决定\晚上\去\看电影\，匆匆\来到\电影院\，刚好\碰到\以前的\老同事\。

probe word：王成（T）黄静（F）

making judgment：王成在电影院碰到以前的老同事（T）

10. 李剑\渴得\嗓子\冒烟\，赶忙\走进\路边的\杂货点\，想买\瓶\汽水\解解渴\。

probe word：李剑（T）孙武（F）

making judgment：李剑发现路边的杂货点冒烟了（F）

11.朱君\打算\去\练\口语\，一大早\就\来到\英语角\，跟\一个\外教\聊了\起来\。

probe word：朱君（T）王宁（F）

making judgment：朱君从来不敢跟人讲英语（F）

12. 张灵\走进\自习室\，找了\个\靠窗的\位置\，顺手\帮\同学\占了\个\座位\。

probe word：张灵（T）陈慧（F）

making judgment：张灵在自习室帮同学占了个座位（T）

13.一下班\徐刚\就\冲出\办公室\，很快\就\来到\附近的\饭店\，等\朋友\一起\吃\晚饭\。

probe word：徐刚（T）贺兵（F）

making judgment：徐刚几乎从不在家吃晚饭（F）

14. 王锋\听到\一声\巨响\，便\迅速\冲出\猫耳洞\，看到\战友\倒在\前面的\山坡上\。

probe word：王锋（T）张岩（F）

making judgment：王锋不幸倒在了猫耳洞旁（F）

15. 孙琳\周末\去逛街\，半天\下来\觉得\又累又饿\，决定\找\个\地方\歇一歇\。

probe word：孙琳（T）王玲（F）

making judgment：孙琳逛街逛得又累又饿（T）

16. 林红\打开\客厅的\灯\，懒洋洋\地\躺在\沙发\上\，觉得\自己\都\快散架了\。

probe word：林红（T）夏青（F）

making judgment：林红以为沙发快散架了（F）

17. 吴岩\发现\外面\下起了\大雨\，连忙\冲到\阳台上\，收拾\晒在\外面的\衣物\。

probe word：吴岩（T）赵刚（F）

making judgment：吴岩被大雨淋的全身都湿透了（F）

18. 天刚亮\王钢\就\起了床\，发现\时间\其实\不早了\，便\飞快地\冲向\汽车站\。

probe word：王钢（T） 李平（F）

making judgment：王钢非常担心自己迟到（T）

19. 星期天\王丽\在\图书馆\看书\，直到\要关门时\，才\不舍地\放下\手中的书\。

probe word：王丽（T）吴为（F）

making judgment：王丽把图书馆的门关上了（F）

20. 上午\刘勇\在寝室\打电脑游戏\，玩得\开心\极了\，看样子\下午\会\继续\打下去\。

probe word：刘勇（T）李冰（F）

making judgment：刘勇打了一上午的电脑游戏（T）

21. 王华\今天\约了\朋友\出去\玩\，兴冲冲\到达\公园\后\，发现\忘了\带相机\。

probe word：王华（T）陈艳（F）

making judgment：王华在公园给朋友拍了许多照片（F）

22. 赵勇\听见\下课\铃声\，马上\拿起\装碗\的袋子\，直盯着\还在\滔滔不绝的\老师\。

probe word：赵勇（T）何川（F）

making judgment：赵勇希望老师不要拖堂（T）

23. 回到家里\刘伟\打开\电脑\，进入\自己\的\邮箱\，发现\邮箱里\全是\垃圾邮件\。

probe word：刘伟（T）赵明（F）

making judgment：刘伟的邮箱里全是垃圾邮件（T）

24. 王红\周末\上\函授课\，连续\讲了\两个\整天\，最后\觉得\喉咙\都\快起火了\。

probe word：王红（T）曾强（F）

making judgment：王红上课上得很辛苦（T）

Materials of Experiment 2b

1. 王锋\突然\听到\一声巨响\，\迅速\冲出\潜伏的\猫耳洞\，\看到\战友\倒在\前面的\山坡\上\，\浑身是血\艰难地\挪动着，\便\毫不犹豫地\冲\了\上去\。

probe word：王锋（T）战友（T）李平（F）

making judgment：王锋奋力抢救受伤的战友（T）

2. 林冰\放学后\去\喂\鸭子\，把\鸭子\赶到\村旁\的\池塘里\，\突然\看见\几个\小孩\赤身露体\，一起\在\池塘\追逐打闹\，大声\叫\他们\小心\。

probe word：林冰（T）小孩（T）杨艺（F）

making judgment：林冰看见几个小孩在池塘里玩耍（T）

3. 晚饭后\邓义\准备\出去\走走\，刚刚\走出\家门\，就\遇到\前来\探访的\亲戚\，拎着\一个\鼓鼓囊囊\的\袋子\，心想\又\有\什么\事\了\。

probe word：邓义（T）亲戚（T）张灵（F）

making judgment：邓义见到前来探访的亲戚（T）

4.李明\正在\图书馆\安静地\读书\，突然\听见\身边\有声音\，仔细一看\原来\有人\鬼鬼祟祟\四处张望\，个子\不高\蒙着\头套\，不禁\暗想\这人\到底\要\干什么\。

probe word：李明（T）有人（T）刘伟（F）

making judgment：有人影响李明的读书（T）

5. 张莉\星期天\去\外婆\家\玩\，见到\一个\熟悉\的\身影\，发现\原来\是\很久不见的\朋友\，长得\完全\都\变了\模样\，马上\上前\去\打招呼\。

probe word：张丽（T）朋友（T）钱宁（F）

making judgment：张莉的朋友长得还是老样子（F）

6.李平\今天\参加\职称\考试\，满怀信心\步入\考场\，突然\听到\老师\喝令\他站住\，气势汹汹\地\叫\他\拿出\证件\，顿时\觉得\非常\反感\。

probe word：李平（T）老师（T）张宏（F）

making judgment：李平觉得监考老师非常关心他（F）

7. 杨辉\晚饭后\到\湖边\散步\，突然\听到\有人\喊\"救命"\的\声音\，见\是\一个\小孩\掉进\了\湖里\，正在\痛苦地\挣扎着\，便\二话不说\马上\跳进\湖中\。

probe word：杨辉（T）小孩（T）王玲（F）

making judgment：杨辉毫不犹豫地抢救落水儿童（T）

8. 陈娜\星期五\晚上\去\逛街\，刚刚\准备\过马路\，看见\一个\老奶奶\拿着\很多\东西\，步履蹒跚地\也要\过\马路\，赶忙\上前\去帮忙\。

probe word：陈娜　（T）老奶奶（T）张华（F）

making judgment：陈娜装着没有看见过马路的老奶奶（F）

9. 王萍\周末\到\书店\买书\，高高兴兴\到\了\购书中心\，看见\很多\小学生\，穿着\校服\戴着\红领巾\，心想\今天\莫非\有\什么活动\。

probe word：王平 （T）小学生（T）朱军（F）

making judgment：王萍是一名戴着红领巾的小学生（F）

10.张成\正在\静静地\睡觉\，突然\听到\急促的\敲门声\，开门一看\是\一位\很久不见的\叔叔\，满头大汗\神色慌张\，忙问\出了\什么\事情\。

probe word：张成 （T）叔叔（T）王永（F）

making judgment：张成见到一位很久不见的叔叔（T）

11. 张宇\刚刚\走到\小区\大门口\，突然\听到\咣铛一声\，发现\不远处\一个小孩\从\地上\爬起来\，正\用力\拍打着\书包\，心想\肯定\是\摔跟头\了\。

probe word：张宇（T）小孩（T）叶平（F）

making judgment：张宇一不小心摔了个跟头（F）

12. 李红\晚上\在家里\做\作业\，忽然\听见\楼下\草地上\有\声音\，原来\是\邻居\在\练武 \，把\一套拳\打得\如\行云流水\一般\，不禁\暗暗\叫好\。

probe word：李红 （T）邻居 （T）王永（F）

making judgment：李红的武术工夫特别好（F）

13. 方明\走路\去\表哥\家里\，过了一会\发现\自己\迷路\了\，好不容易\见到\一位\警察\，非常\热心地\回答\问题\，心里\不禁\涌上\感激之情\。

probe word：方明（T）警察（T）高兰（F）

making judgment：警察热心地为方明指路（T）

14. 张军\正在\校园\里\画着\写生\，耳边\传来\银铃般的\笑声\，原来是\一群\少女\在\开心地\玩要\，长得\都\非常\活泼\可爱\，不禁\想把\这一幕\记录下来\。

probe word：张军（T）少女（T）孙伟（F）

making judgment：少女们的活泼感染了张军（T）

15. 李宁\第一次\参加\跆拳道\比赛\，忐忑不安\来到\比赛场地\，见到了\自己的\对手\，长得\高高大大的\，心里\感到\十分紧张\。

probe word：李宁 （T）对手 （T）徐征（F）

making judgment：李宁对即将开始的比赛很有信心（F）

16. 刘芳\星期一\终于\转到了\新的\学校\，一大早\便\高兴地\来到\学校\，见到了\新的\老师\，非常\和蔼可亲\，打心眼里\觉得\喜欢\。

probe word：刘芳（T）老师（T）王双（F）

making judgment：刘芳非常喜欢自己的新老师（T）

17. 赵华\正\参加\同事的\生日晚会\，见到了\很多熟人\，留意到\一位\很特别的\女生\，穿着\朴素的\校服\，总\觉得\有几分\眼熟\。

probe word：赵华（T）女生（T）金立（F）

making judgment：赵华感到自己似乎认识参加晚会的女生（T）

18. 王红\晚上\坐车\去\看\演唱会\，高高兴兴地\来到\体育场，\终于\见到\自己\心仪已久的\歌星\，长得\青春靓丽\英俊潇洒\，甚至\忘记\自己\姓什么了\。

probe word：王红 （T）歌星 （T）韩建（F）

making judgment：王红是一名长得非常漂亮的歌星（F）

19. 陈立\放下\电话\，匆匆\赶往\校门口\，远远\看见\母亲\就\站在\校门旁\，跟\门卫\正\比划着什么\，不由得\赶快\冲了\过去\。

probe word：陈立（T）母亲（T）刘芳（F）

making judgment：母亲的到来让陈力非常激动（T）

20. 刘刚\刚要\过马路\，突然\看见\有\一个\身影\走过来\，猛然\发现\是\一位\警察\，不停地\挥手示意\，于是\赶紧\停住\脚步\。

probe word：刘刚（T ）警察（T ） 王炎（F）

making judgment：刘刚的挥手使保安停了下来（F）

21. 黄丽\早上\在\等\校车\，见到\马路边的\店铺\里\有人\打招呼\，认出\是\同学\吴霞\，抱怨说\今天\校车\坏了\，心想\今天\要\打\的士\去\学校\了\。

probe word：黄丽（T ）吴霞（T ）丁波（F）

making judgment：吴霞告诉黄丽校车出了故障（T）

22. 张红\上午\去\菜市场\买菜\，在\市场\门口\看见\许多人\围成\一堆\，走近后\发现\是\一个\乞丐\躺在\地上\，正用\粉笔\写着什么\，禁不住暗暗好奇\。

probe word：张红（T）乞丐（T）吴非（F）

making judgment：张红非常同情乞丐（F）

23. 李勇\坐在\公车上\昏昏欲睡\，隐约\感到\提包\不见了\，抬头一看\，有人\正\把\手\缩回去\，并\装着\若无其事的\样子\，于是\用力一拽\把\包\夺\了\回来\。

probe word：刘刚（T）有人（T）李锐（F）

making judgment：李勇没有把东西丢失放在心上（F）

24. 王娟\正\照顾\生病的\妈妈\，突然\听到\敲门的\声音\，见是隔壁的\阿姨\，拿着\一篮\新鲜的\水果\，忙请\她\进\屋里\。

probe word：王娟（T）阿姨（T）周春（F）

making judgment：隔壁的阿姨给王娟送来了水果（T）

Experiment Materials of Experiment 3

(1) the situation described by the first two clauses in the active voice was consistent (the first-mention priority)

1、打官司　律师告诉法官，不能接受对方开出的条件。

probe word：律师（T）法官（T）客户（F）

2、抓坏人　　警察迅速抓住小偷，将其扭送到公安机关。

probe word：警察（T）小偷（T）水手（F）

3、在课堂上　　老师狠狠批评了班上最调皮的学生，警告他不要再犯错误。

probe word：老师（T）学生（T）职员（F）

4、在图书馆　　门卫拦住学生，要求其出示学生证。

probe word：门卫（T）学生（T）厨师（F）

5、在办公室　　领导找来小张谈话，询问最近工作情况。

probe word：领导（T）小张（T）教授（F）

6、在画室里　老师叫小明到跟前，认真地看着他的素描习作本。

probe word：老师（T）小明（T）船员（F）

7、开会　连长在会上通知全体战士，将不定期进行内务检查。

probe word：连长（T）战士（T）民工（F）

8、跳舞　男孩注视着舞池里那位漂亮的女孩，希望与其一起跳舞。

probe word：男孩（T）女孩（T）保姆（F）

9、在商店　顾客告诉店员，要买一双大码的拖鞋。

probe word：顾客（T）店员（T）球员（F）

10、旅游　导游提醒游客，要求其照顾好自己的小孩。

probe word：导游（T）游客（T）专家（F）

11、吃饭　母亲告提醒小华，要求他吃饭时别讲话。

probe word：母亲（T）小华（T）叔叔（F）

12、在火车上　乘警怀疑挤上车的矮个子乘客，要求他出示火车票。

probe word：乘警（T）乘客（T）工人（F）

13、购书　经理遗憾地告诉顾客，不能给新书打九折优惠。

probe word：经理（T）顾客（T）班长（F）

14、送信　邮递员叮嘱开门的小孩，提醒她不要把信弄丢了。

probe word：邮递员（T）小孩（T）司机（F）

15、哄孩子　妈妈抱起摇篮里大声哭闹的婴儿，让其慢慢安静下来。

probe word：妈妈（T）婴儿（T）客人（F）

16、在医院　护士拦住等候在急诊室外的家属，警告他不要在医院吸烟。

probe word：护士（T）家属（T）房东（F）

17、在公司　经理高度赞扬了公司本年度最优秀的员工，希望她以后做得更好。

probe word：经理（T）员工（T）农民（F）

18、理发店　理发师轻轻拍了一下顾客，提醒他不要总是动来动去。

probe word：理发师（T）顾客（T）机师（F）

19、在家里　丈夫惊讶地瞪着妻子，不知道她为什么穿得这付模样。

probe word：丈夫（T）妻子（T）父亲（F）

20、送别　群众夹道欢送军人，感谢他们帮助抵御百年不遇的洪水。

probe word：群众（T）军人（T）演员（F）

21、演奏会　听众疑惑地看着钢琴家，不知道此刻他为什么弹这首曲子。

probe word：听众（T）钢琴家（T）诗人（F）

22、准备手术　医生紧张地盯着被麻醉的病人，看他是否进入深度昏迷。

probe word：医生（T）病人（T）奶奶（F）

23、抓小偷　保安把刚得手的小偷死死抓住，喝令他交出赃物。

probe word：保安（T）小偷（T）　翻译（F）

24、在幼儿园　保育员细心照看每个幼儿，保证他们吃好睡好。

probe word：保育员（T）幼儿（T）老人（F）

25、迟到　老板严厉训斥了下属，要求他保证下次不再迟到。

probe word：老板（T）下属（T）导师（F）

26、在机场　即将远行的女儿紧紧地搂着母亲，叮嘱她要多注意身体。

probe word：女儿（T）母亲（T）博士（F）

(2) the situation described by the first two clauses in the passive voice was consistent

1、打官司　法官被律师告之，不能接受对方开出的条件。

probe word：原告（T）律师（T）客户（F）

2、抓坏人　小偷被警察迅速抓住，将其扭送到公安机关。

probe word：警察（T）小偷（T）水手（F）

3、在课堂上　班上最调皮的学生被老师狠狠批评，警告他不要再犯错误。

probe word：老师（T）学生（T）职员（F）

4、在图书馆　学生被门卫拦住，要求其出示学生证。

probe word：门卫（T）学生（T）厨师（F）

5、在办公室　小张被领导找来谈话，询问最近工作情况。

probe word：领导（T）小张（T）教授（F）

6、在画室里　小明被老师叫到跟前，认真地看着他的素描习作本。

probe word：老师（T）小明（T）船员（F）

7、开会　在会上全体战士被连长通知，将不定期进行 内务检查。

probe word：连长（T）战士（T）民工（F）

8、跳舞　舞池里那位漂亮的女孩被男孩注视着，希望与其一起跳舞。

probe word：男孩（T）女孩（T）保姆（F）

9、在商店　　店员被顾客告之，要买一双大码的拖鞋。

probe word：顾客（T）店员（T）球员（F）

10、旅游　　游客被导游提醒，要求其照顾好自己的小孩。

probe word：导游（T）游客（T）专家（F）

11、吃饭　　小华被母亲提醒，要求他吃饭时别讲话。

probe word：母亲（T）小华（T）叔叔（F）

12、在火车上　　挤上车的矮个子乘客受到乘警怀疑，要求他出示火车票。

probe word：乘警（T）乘客（T）工人（F）

13、购书　　顾客被经理遗憾地告之，不能给新书打九折优惠。

probe word：经理（T）顾客（T）班长（F）

14、送信　　开门的小孩被邮递员叮嘱，提醒她不要把信弄丢了。

probe word：邮递员（T）小孩（T）司机（F）

15、哄孩子　　摇篮里大声哭闹的婴儿被妈妈抱起，让其慢慢安静下来。

probe word：妈妈（T）婴儿（T）客人（F）

16、在医院　　等候在急诊室外的家属被护士拦住，警告他不要在医院吸烟。

probe word：护士（T）家属（T）房东（F）

17、在公司　　公司本年度最优秀的员工受到经理高度赞扬，希望她以后做得更好。

probe word：经理（T）员工（T）　农民（F）

18、理发店　　顾客被理发师轻轻拍了一下，提醒他不要总是动来动去。

probe word：理发师（T）顾客（T）机师（F）

19、在家里　　妻子被丈夫惊讶地瞪着，不知道她今天为什么穿得这付模样。

probe word：丈夫（T）妻子（T）父亲（F）

20、送别　　军人被群众夹道欢送，感谢他们帮助抵御百年不遇的洪水。

probe word：群众（T）军人（T）演员（F）

21、演奏会　　钢琴家被听众疑惑地看着，不知道此刻他为什么弹这首曲子。

probe word：钢琴家（T）听众（T）诗人（F）

22、准备手术　　被麻醉的病人被医生紧张地盯着，看他是否进入深度昏迷。

probe word：医生（T）病人（T）奶奶（F）

23、抓小偷　　刚得手的小偷被保安死死抓住，喝令他交出赃物。

probe word：保安（T）小偷（T）翻译（F）

24、幼儿园里　　幼儿被保育员 细心照看，保证他们吃好睡好。

probe word：保育员（T）幼儿（T）老人（F）

25、迟到　　下属被老板严厉训斥，要求他保证下次不再迟到。

probe word：老板（T）下属（T）导师（F）

26、在机场　　母亲被即将远行的女儿紧紧地搂着，叮嘱她要多注意身体。

probe word：女儿（T）母亲（T）博士（F）

(3) the situation described by the first two clauses in the active voice was inconsistent (the first-mention priority)

1、在课堂上　　律师告诉法官，不能接受对方开出的条件。

probe word：法官（T）律师（T）客户（F）

2、说谎　　警察迅速抓住小偷，将其扭送到公安机关。

probe word：警察（T）小偷（T）水手（F）

3、看病　　老师狠狠批评了班上最调皮的学生，警告他不要再犯错误。

probe word：老师（T）学生（T）职员（F）

4、在餐厅　　门卫拦住学生，要求其出示学生证。

probe word：门卫（T）学生（T）厨师（F）

5、田径场上　　领导找来小张谈话，询问最近工作情况。

probe word：领导（T）小张（T）教授（F）

6、吃饭　　老师叫小明到跟前，认真地看着他的素描习作本。

probe word：老师（T）小明（T）船员（F）

7、赛跑　　连长在会上通知全体战士，将不定期进行 内务检查。

probe word：连长（T）战士（T）民工（F）

8、在商店　　男孩注视着舞池里那位漂亮的女孩，希望与其一起跳舞。

probe word：男孩（T）女孩（T）保姆（F）

9、在幼儿园　　顾客告诉店员，要买一双大码的拖鞋。

probe word：顾客（T）店员（T）球员（F）

10、上课　　导游提醒游客，要求其照顾好自己的小孩。

probe word：导游（T）游客（T）专家（F）

11、开会　　母亲提醒小华，要求他吃饭时别讲话。

probe word：母亲（T）小华（T）叔叔（F）

12、在公司　　乘警怀疑挤上车的矮个子乘客，要求他出示火车票。

probe word：乘警（T）乘客（T）工人（F）

13、在餐馆　　经理遗憾地告诉顾客，不能给新些书打九折优惠。

probe word：经理（T）顾客（T）班长（F）

14、购书　　邮递员叮嘱开门的小孩，提醒她不要把信弄丢了。

probe word：邮递员（T）小孩（T）司机（F）

15、训练场上　　妈妈抱起摇篮里大声哭闹的婴儿，让其慢慢安静下来。

probe word：妈妈（T）婴儿（T）客人（F）

16、在火车上　　护士拦住等候在急诊室外的家属，警告他不要在医院吸烟。

probe word：护士（T）家属（T）房东（F）

17、看电影　　经理高度赞扬了公司本年度表现最优秀的员工，希望她以后做得更好。

probe word：经理（T）员工（T）农民（F）

18、在医院　　理发师轻轻拍了一下顾客，提醒他不要总是动来动去。

probe word：理发师（T）顾客（T）机师（F）

19、在法庭上　　丈夫惊讶地瞪着妻子，不知道她为什么穿得这付模样。

probe word：丈夫（T）妻子（T）父亲（F）

20、在战场　　群众/夹道/欢送/军人/，/感谢/他们/帮助抵御百年不遇的洪水。

probe word：群众（T）军人（T）演员（F）

21、足球赛　　听众/疑惑地/看着/钢琴家/，/不知道/此刻/他/为什么/弹/这首/曲子/。

probe word：听众（T）钢琴家（T）诗人（F）

22、下象棋　　医生紧张地盯着被麻醉的病人，看他是否进入深度昏迷。

probe word：医生（T）病人（T）奶奶（F）

23、玩游戏　　保安把刚得手的小偷死死抓住，喝令他交出赃物。

probe word：保安（T）小偷（T）翻译（F）

24、开会　　保育员细心照看每个幼儿，保证他们吃好睡好。

probe word：保育员（T）　幼儿（T）老人（F）

25、参观　　老板严厉训斥了下属，要求他保证下次不再迟到。

probe word：老板（T）下属（T）导师（F）

26、在商场　　即将远行的女儿紧紧地搂着母亲，叮嘱她要多注意身体。

probe word：女儿（T）母亲（T）博士（F）

(4) the situation described by the first two clauses in the passive voice was inconsistent

1、在课堂上　　法官被律师告之，不能接受对方开出的条件。

probe word：法官（T）律师（T）客户（F）

2、说谎　　小偷被警察迅速抓住，将其扭送到公安机关。

probe word：警察（T）小偷（T）水手（F）

3、看病　　班上最调皮的学生被老师狠狠批评，警告他不要再犯错误。

probe word：老师（T）学生（T）职员（F）

4、在餐厅　　学生被门卫拦住，要求其出示学生证。

probe word：门卫（T）学生（T）厨师（F）

5、田径场上　　小张被领导找来谈话，询问最近工作情况。

probe word：领导（T）小张（T）教授（F）

6、吃饭　　小明被老师叫到跟前，认真地看着他的素描习作本。

probe word：老师（T）小明（T）船员（F）

7、赛跑　　在会上全体战士被连长通知，将不定期进行内务检查。

probe word：连长（T）战士（T）民工（F）

8、在商店　　舞池里那位漂亮的女孩被男孩注视着，希望与其一起跳舞。

probe word：男孩（T）女孩（T）保姆（F）

9、在幼儿园　　店员被顾客告之，要买一双大码的拖鞋。

probe word：顾客（T）店员（T）球员（F）

10、上课　　游客被导游提醒，要求其照顾好自己的小孩。

probe word：导游（T）游客（T）专家（F）

11、开会　　小华被母亲提醒，要求他吃饭时别讲话。

probe word：母亲（T）小华（T）叔叔（F）

12、在公司　　挤上车的矮个子乘客被乘警怀疑，要求他出示火车票。

probe word：乘警（T）乘客（T）工人（F）

13、在餐馆　　顾客被经理遗憾地告之，不能给新书打九折优惠。

probe word：经理（T）顾客（T）班长（F）

14、购书　开门的小孩被邮递员叮嘱，提醒她不要把信弄丢了。

probe word：邮递员（T）小孩（T）司机（F）

15、训练场上　摇篮里大声哭闹的婴儿被妈妈抱起，让其慢慢安静下来。

probe word：妈妈（T）婴儿（T）客人（F）

16、在火车上　等候在急诊室外的家属被护士拦住，警告不要在医院吸烟。

probe word：护士（T）家属（T）房东（F）

17、看电影　公司本年度最优秀的员工被经理高度赞扬，希望她以后做得更好。

probe word：经理（T）员工（T）农民（F）

18、在医院　顾客被理发师轻轻拍了一下，提醒他不要总是动来动去。

probe word：理发师（T）顾客（T）机师（F）

19、在法庭上　妻子被丈夫惊讶地瞪着，不知道她今天为什么穿得这付模样。

probe word：丈夫（T）妻子（T）父亲（F）

20、在战场　军人/被/群众/夹道/欢送，/感谢/他们/帮助抵御百年不遇的洪水。

probe word：群众（T）军人（T）演员（F）

21、足球赛　钢琴家/被/听众/疑惑地/看着/，/不知道/此刻/他/为什么/弹/这首/曲子/。

probe word：钢琴家（T）听众（T）诗人（F）

22、下象棋　被麻醉的病人被医生紧张地盯着，看他是否进入深度昏迷。

probe word：医生（T）病人（T）奶奶（F）

23、玩游戏　刚得手的小偷被保安死死抓住，喝令他交出赃物。

probe word：保安（T）小偷（T）翻译（F）

24、开会　幼儿被保育员细心照看，保证他们吃好睡好。

probe word：保育员（T）幼儿（T）老人（F）

25、参观　下属被老板严厉训斥，要求他保证下次不再迟到。

probe word：老板（T）下属（T）导师（F）

26、在商场　母亲被即将远行的女儿紧紧地搂着，叮嘱她要多注意身体。

probe word：女儿（T）母亲（T）博士（F）
